# Morpho-Physiological Traits and Functional Markers Based Molecular Dissection of Heat-Tolerance in Urdbean

**DOI:** 10.3389/fpls.2021.719381

**Published:** 2021-09-29

**Authors:** Debjyoti Sen Gupta, Partha S. Basu, J. Souframanien, Jitendra Kumar, P. Dhanasekar, Sanjeev Gupta, Muthaiyan Pandiyan, S. Geetha, P. Shanthi, Vaibhav Kumar, Narendra Pratap Singh

**Affiliations:** ^1^Division of Crop Improvement, ICAR-Indian Institute of Pulses Research, Kanpur, India; ^2^All India Coordinated Research Project on Mungbean, Urdbean, Lentil, Lathyrus, Rajmash, and Fieldpea, ICAR-Indian Institute of Pulses Research, Kanpur, India; ^3^Division of Basic Sciences, ICAR-Indian Institute of Pulses Research, Kanpur, India; ^4^Nuclear Agriculture & Biotechnology Division, Bhabha Atomic Research Centre, Mumbai, India; ^5^National Pulses Research Centre, Vamban, India; ^6^Division of Biotechnology, ICAR-Indian Institute of Pulses Research, Kanpur, India

**Keywords:** *Vigna mungo*, heat tolerance, abiotic stress, membrane stability, electron transport rate, heat susceptibility index, chlorophyll fluorescence, molecular markers

## Abstract

Urdbean (*Vigna mungo* L. Hepper) is one of the important pulse crops. Its cultivation is not so popular during summer seasons because this crop is unable to withstand excessive heat stress beside lack of humidity in the atmosphere. Therefore, a panel of 97 urdbean diverse genotypes was assessed for yield under stress and non-stress conditions with an aim to identify heat tolerant genotypes. This study identified 8 highly heat tolerant and 35 highly heat sensitive genotypes based on heat susceptibility index. Further, physiological and biochemical traits-based characterization of a group of six highly heat sensitive and seven highly heat tolerant urdbean genotypes showed genotypic variability for leaf nitrogen balance index (NBI), chlorophyll (SPAD), epidermal flavnols, and anthocyanin contents under 42/25°C max/min temperature. Our results showed higher membrane stability index among heat tolerant genotypes compared to sensitive genotypes. Significant differences among genotypes for ETR at different levels of PAR irradiances and PAR × genotypes interactions indicated high photosynthetic ability of a few genotypes under heat stress. Further, the most highly sensitive genotype PKGU-1 showed a decrease in different fluorescence parameters indicating distortion of PS II. Consequently, reduction in the quantum yield of PS II was observed in a sensitive one as compared to a tolerant genotype. Fluorescence kinetics showed the delayed and fast quenching of Fm in highly heat sensitive (PKGU 1) and tolerant (UPU 85-86) genotypes, respectively. Moreover, tolerant genotype (UPU 85-86) had high antioxidant activities explaining their role for scavenging superoxide radicals (ROS) protecting delicate membranes from oxidative damage. Molecular characterization further pinpointed genetic differences between heat tolerant (UPU 85-86) and heat sensitive genotypes (PKGU 1). These findings will contribute to the breeding toward the development of heat tolerant cultivars in urdbean.

## Introduction

Urdbean (*Vigna mungo* L. Hepper) is a popular food legume grown in many Asian countries including India, Pakistan, Myanmar, Bangladesh, Thailand, and China. India is the largest producer and consumer of urdbean. It produces about 3.36 million tons of urdbean (Project Coordinator’s Report, 2019-2020) and imports another 0.5 million tons from other urdbean growing countries, particularly from Myanmar. During 2014-2015, Myanmar produced 1.51 million tons of urdbean that is locally known as black matpe bean. Nutritionally, urdbean is dense with protein (21-28%), dietary fiber (161-187 g/kg), iron (16-255 mg/kg), zinc (5-134 mg/kg), and other micronutrients like other pulses (Chitra et al., 1996; Sen Gupta et al., 2020). Therefore, its nutrient-dense profile has encouraged an introduction to many developed countries including the United States, Russia, and European nations as a potential pulse crop (Sen Gupta et al., 2020).

Urdbean is grown in different ecologies and seasons across the growing regions. In India, it is grown mainly in the rainy season (July-October) and in the southern part it is also cultivated as a winter season crop (November to February). However, its cultivation is not wide in the summer season due to excessive heat stress and a lack of humidity in the atmosphere. Thus, availability of heat tolerant cultivars can bring more areas under urdbean cultivation. Previously, genetic variability for heat tolerance was reported in many food legumes (Sita et al., 2017), but it is not yet explored in urdbean. It is a warm season food legume, which requires 25-35°C temperature along with high humidity for its normal growth and development. However, prevailing high temperature (>40°C) during flowering results in deformation of flower parts or flower drop leading to negative impact on yield. Similarly, in mungbean, higher temperatures of >38/25°C (day and night, respectively) markedly affected the yield under summer-season cultivation (Nayyar et al., 2017).

The effect of heat stress results in drastic yield losses due to pollen or ovule inactivity, flower abortion, and even post-fertilization impaired growth and development of embryo or seed in many pulses (Sita et al., 2017). Moreover, the current climate change scenario also leads to abrupt changes in mean temperature. Therefore, breeding of heat tolerant urdbean varieties becomes more relevant under such situations. Urdbean is a close relative of mungbean, which is extensively cultivated in identical ecologies. In this crop as well as in another *Vigna* pulse crop, cowpea, sources of heat tolerance have already been identified (Ehlers and Hall, 1998; Basu et al., 2019).

Knowledge of key traits imparting heat tolerance can help to improve the grain yield of urdbean (Scafaro et al., 2010). Therefore, physio-biochemical mechanisms underlying these key traits are essential to screen large numbers of germplasm at critical temperature under both field and controlled conditions (Gaur et al., 2019). In several other crops, various physiological traits such as photosynthetic activity, membrane stability, pollen viability, and phenolic compounds have been used to identify heat tolerant genotypes (Allakhverdiev and Murata, 2004; Asseng et al., 2015; Sita et al., 2017) and genetic variability has been reported for key physiological traits under heat stress conditions (Challinor et al., 2007).

Urdbean is a highly photothermo-sensitive crop. Therefore, its yield potential varies across locations due to variable daylength and thermal regimes. Thus, minimizing the genotype × environment interactions can help to achieve stable yield of urdbean. The high temperature stress above the threshold across the locations during the summer season could be the compounding effects of both heat and photosensitivity. One of the strategies for selecting photo-thermo insensitive lines is to evaluate different genotypes at multi-locations having varying daylength and thermal regimes. As a result, genotypes having stable yield across the locations could be identified as putative photo-thermo insensitive lines. This strategy should be made to screen thermo-tolerant lines from the panel of photo-thermo insensitive lines so that widely adapted stable heat tolerant lines could be identified having less influence of photo-thermoperiods. In the present investigation, this approach has been followed to identify contrasting genotypes having a high level of tolerance or sensitivity to high temperature.

Knowledge of genetics underlying key traits imparting heat tolerance helps the breeder to make genetic improvements more precisely. In recent years, molecular markers helped to decipher the genetics of complex key morpho-physiological traits imparting heat tolerance in several crops (Argyris et al., 2008; Roy et al., 2011; Paliwal et al., 2012). However, in urdbean, use of molecular markers for mapping and characterization of traits related to heat tolerance is poorly understood. Currently, simple sequence repeats (SSRs) and single nucleotide polymorphisms (SNPs) are available for molecular characterization in urdbean (Raizada and Souframanien, 2019; Souframanien et al., 2020; Pootakham et al., 2021). Hence, this experiment was designed with the following objectives: (i) to evaluate a set of urdbean genotypes under field conditions with natural heat stress conditions (flowering and podding stage coincides with high temperature), and to compare it with normal field conditions (comparatively less exposure to high temperature during flowering and podding), (ii) to precisely phenotype selected contrasting heat tolerant and sensitive genotypes for different physiological and biochemical traits, and (iii) to characterize heat tolerant and sensitive genotypes with heat-related genic markers.

## Materials and Methods

### Plant Materials

Plant materials comprised of 97 urdbean genotypes, which were grown during the summer season of 2016 at the Main Research Farm of Indian Institute of Pulses Research (IIPR), Kanpur (26.28°N and 80.21°E), and National Pulses Research Centre of TNAU, Vamban (10.20°N, 78.50° E) in India. The tested urdbean genotypes comprised of germplasm, breeding lines, and cultivars of diverse origins (Supplementary File 1). Maturity duration for these genotypes ranged from 70 to 75 days. The field experiments were grown in augmented- randomized complete block (RCB) design. Three checks (Uttara, Shekhar-2, IPU 02-43) were replicated with randomization in each one of the five blocks. Each plot consisted of double rows of 4 m length. Rows were spaced 30 cm apart and interplant distance was 10 cm. Two trials were conducted at each location and based on meteorological data and average yield of trial one was designated as “stress environment (SE)” and another was named as “non-stress environment (NSE).” Standard practices were followed to raise the rainfed crop excluding one pre-sown irrigation.

### Heat Susceptibility Index

Heat susceptibility index (HSI) for each individual urdbean genotype was calculated using the equation by Fischer and Maurer (1978): HSI = (1-Yh/Y)/(1-Xh/X) where Yh and Y are the phenotypic means (Yield) for each genotype under heat stress and non-heat stress conditions, respectively, and Xh and X are the phenotypic means (Yield) for all lines under heat stressed and non-heat stress conditions, respectively.

### Meteorological Data Collection

Weather data from Kanpur and Vamban locations were recorded throughout the growing period by the respective meteorological observatories present in both places.

### Physiological Characterization of Heat Tolerant and Sensitive Genotypes

#### Plant Samples Under Controlled Environment

Seeds of selected contrasting urdbean genotypes were obtained from the urdbean breeding program of IIPR, Kanpur. Seeds were surface sterilized with 70% ethanol for 5 min, followed by treatment with 1% sodium hypochlorite (v/v) for 3 min. The sterilized seeds were rinsed 3 times with sterile Milli-Q (Merck Millipore, Germany) water under aseptic conditions and soaked overnight at room temperature.

The sterilized seeds were sown in cocopit–vermicompost–soil mixture (3:1:1 ratio) and irrigated with Hoagland solution. The plants were raised under a controlled environment chamber (Hi-point, Taiwan) and maximum minimum temperature 40/25°C with 14-h photoperiod was maintained. The light sources were RGB LED (Red-Green-Blue-White) having an irradiance level of 460 μmol photons m^–2^s^–1^ and relative humidity 80%. The required moisture and fertility of the soil compost was ensured by irrigating with water or Hoagland solution at regular intervals.

#### Nitrogen Balance Index, Chlorophyll, Anthocyanin, and Flavanols Contents

The physiological status of selected plant leaves was determined using a hand-held device DUALEX leaf clip device (Force-A, France), which enabled comparative values of leaf chlorophyll (leaf greenness) content (Chl), epidermal flavanols (Flv), nitrogen balance index (NBI), and anthocyanin content of leaves subjected to heat stress in a sensor-controlled chamber consistently maintained at 40/25°C (maximum/minimum).

##### Measurement of chlorophyll

DUALEX measures the chlorophyll content of a leaf based on the transmittance ratio at two different wavelengths. One in the far-red absorbed by chlorophyll and one in the near-infrared as a reference. The leaf chlorophyll content can rapidly and accurately be assessed from light transmittance. A first wavelength very close to the red quantifies the chlorophyll and a second in the near-infrared can take into account the effects of leaf structure.

Chlorophyll index = (Near-infrared transmittance – Red transmittance)/(Red transmittance)

##### Measurements of polyphenols (flavanols) and anthocyanin

DUALEX measures flavanols and anthocyanins content of the leave’s epidermis based on differential ratio of chlorophyll fluorescence. Near-infrared chlorophyll fluorescence is measured under a first reference excitation light not absorbed by polyphenols. It is compared to a second sampling light specific to a particular type of polyphenols (e.g., green for anthocyanins or UV-A for flavanols). Only a fraction of this light reaches the chlorophyll in the mesophyll and can generate near-infrared fluorescence.

Flavanol index = Log (Near-infrared fluorescence excited red/Near-infrared fluorescence excited UV-A)

Anthocyanin index = Log (Near-infrared fluorescence excited red/Near-infrared fluorescence excited green)

##### Differential measurement of fluorescence emitted by chlorophyll

The difference in chlorophyll fluorescence measured in the near-infrared is thus directly proportional to the amount of polyphenols (flavanols) present in the epidermis of the leaf.

##### Measurement of nitrogen balance index

It is the ratio of chlorophyll to flavanol index. Polyphenols, specifically flavanols, are indicators of nitrogen status of plants. Indeed, when a plant is under optimal conditions, it favors its primary metabolism and synthesizes proteins (nitrogen-containing molecules) containing chlorophyll and a few flavanols (carbon-based secondary compounds). On the contrary, in case of nitrogen deficiency, the plant directs its metabolism toward an increased production of flavanols.

#### Membrane Stability

The membrane stability index (MSI) was determined using the electrolyte leakage (EL) method. For keeping uniformity among samples, the well-developed fully expanded fourth leaf from the top of test plants was collected, washed using distilled water, surface dried, and dipped in deionized water at 40°C for 1 h. The electrical conductivity (EC) of tissue leachates was measured using a conductivity meter (Model HI2300, Hanna, United States). The contents were incubated further by dipping the same leaf in deionized water at 100°C for 1 h and EC was measured. The MSI was calculated by the following formula:

MSI = C1/C2, where C1 = EC (EC μS) at 40°C for 1 h and C2 = EC (EC μS) at 100°C for 1 h (Blum and Ebercon, 1981)

#### Fluorescence Image Analysis

Leaf samples of all high temperature (40/25°C; maximum/minimum) grown urdbean genotypes from both groups (heat tolerant and sensitive) were used for chlorophyll fluorescence studies as described by Schreiber and Bilger (1987). High temperature grown genotypes were given hot water heat shock at 43°C for 1 h and thereafter stressed leaves were dark-adapted for 10 min in a temperature-controlled chamber and image analysis was conducted. Photosynthetic response between the tolerant and sensitive lines was assessed using a fluorescence imaging system (Mess & Regeltechnik, Waltz, Germany). The dark-adapted leaves were subjected to 0.05 μmol weak 2 Hz modulated light for 100 μs followed by superimposing saturation light pulses of 4000 μmol m^–2^s^–1^ PAR for 400 ms to obtain quantum yield (Fv/Fm; variable to maximum fluorescence ratio) and fluorescence images were captured. Subsequently, leaves were exposed to actinic light of 200 μmol photons m^–^^2^s^–^^1^ for 2 min for light adaptation. Same saturated pulses were superimposed to obtain quantum yield in light-adapted leaves. Quantum yield (F_*V*_/F_*m*_), maximal fluorescence (F_*m*_), minimum fluorescence (F_0_), and quantum yield of non-regulated energy dissipation [Y(NO)] values were compared between heat tolerant and sensitive genotypes.

#### Photosynthetic Electron Transport Rate

All tested 13 genotypes were pretreated with thermal shock at 43°C for 1 h by inserting leaves in a circulating hot water bath. This temperature was considered detrimental for the photosynthetic membrane and induces disorganization of photosystems and membrane bound electron transport components. Light response of ETR representing the photosynthetic activity of leaves of all tested urdbean genotypes was studied using software ImagingWin (Walz-Imaging System, GmbH, Germany) employing an irradiance range of 200–700 μmolm^–^^2^ s^–^^1^. The light curve and initial fluorescence values (F_0_ and F_*m*_, respectively) of the dark-adapted leaves were used for calculation of ETR (ETR = Quantum yield × PAR × 0.5 × Absorptivity). Absorptivity describes the fraction of incident light, which is absorbed, and 0.5 indicates that only half of the absorbed quanta is distributed to PS II (under steady state conditions). The light curve of an individual selection was obtained with increasing order of irradiance until ETR was light saturated.

#### Fluorescence Parameters During Light–Dark Transition

After measuring the F_0_, F_*m*_ and F_*v*_/F_*m*_ in dark-adapted leaves, the leaves were exposed to actinic light of irradiance 200 μmolm^–^^2^ s^–^^1^ and then saturated light pulse was triggered at every 50 s to obtain F_0_, F_*m*_ and F_*v*_/F_*m*_ in light-adapted leaves until 250 s of illumination. Thereafter, actinic light was switched off and F_0_, F_*m*_ and F_*v*_/F_*m*_ were measured at every 50 s in order to ascertain the restoration of normal F_0_, F_*m*_ and F_*v*_/F_*m*_ in heat-tolerant and sensitive lines during a dark cycle.

In another experiment, high temperature grown contrasting urdbean genotypes were allowed to adapt in the dark for 5 min and thereafter saturated light flash 4000 μmolm^–^^2^ s^–^^1^ was triggered for 100 ms to obtain F_0_ and F_*m*_. Then, leaves were exposed to actinic light 200 μmolm^–^^2^ s^–^^1^ for light adaptation. The light phase was continued until 350 s and then at every 15 s saturated pulse was applied to obtain F_0_ and F_*m*_. Thereafter, leaves were put into a dark phase for adaptation and in a similar manner a saturated pulse was applied at every 15 s to obtain F_0_ and F_*m*_. The only difference between these two events was fluorescence kinetics in light followed by in dark to see the recovery of F_0_ and F_*m*_ in a dark phase.

#### Biochemical Parameters-*in vivo* Visualization of Superoxide Radical and Hydrogen Peroxide

##### *In vivo* visualization of superoxide radical

*In vivo* assay of superoxide radical in the leaf was carried out according to the method of Frahry and Schopfer (2001). Fresh leaf samples were collected and dipped in staining solution for 1 h. The staining solution was composed of 10 mM sodium azide, 100 mM potassium phosphate (pH 7.8), and 0.1 % Nitroblue tetrazolium. After 1 h, leaf samples were bleached by immersing them into boiling ethanol for 15 min. The bleaching solution decolorized the leaves except the dark blue insoluble formazan deposits formed by the reaction of NBT with a superoxide radical. The photographs of the stained samples were captured using a good quality camera for further use.

##### *In vivo* visualization of hydrogen peroxide

The visualization of hydrogen peroxide in the leaf samples was examined using the method of Christensen et al. (1997). The collected leaf samples were washed using double distilled water. The washed samples were dipped into a solution containing 0.1% 3,3′-diaminobenzidine (DAB) dissolved in HCl acidified water (pH 3.8). Then, it was incubated for 16 h to allow the uptake of DAB and its reaction with H_2_O_2_ and peroxidase. The leaf samples were bleached by immersing them in boiling ethanol for 15 min. The photographs of the stained samples were captured using a good quality camera for further use.

### Molecular Characterization

Genic SSR markers were used to characterize eight heat-sensitive (IPU99-200, IC-21001, Shekhar-2, Uttara, PU-19, HPU-120, H-1, PKGU-1) and eight heat-tolerant (UPU-85-86, IPU94-2, IPU-98/36, No. 5/31, PGRU-95014, PGRU-95016, PLU-1, BGP-247) genotypes in the present study. Details of 21 genic-simple sequence repeat (SSR) markers were provided in Table 13.

### DNA Extraction and PCR

DNA was extracted from 1-day-old seedlings by the Dellaporta et al. (1983) method. The SSR primer pairs for sequence-specific markers were designed from leguminous crops having relevance to abiotic stress tolerance (Table 13). PCR reactions were carried out in a 25-μl reaction volume in an Eppendorf Master Cycler (Eppendorf, Hamburg, Germany) with the following composition: 50 ng of genomic DNA, 10 mM Tris-HCl (pH 8.3), 50 mM KCl, 2.5 mM MgCl_2_, 0.08% Non-idet P40, 0.2 mM dNTPs, 1.5 pmoles each of forward and reverse primers, and 0.5 unit Taq DNA polymerase (Fermentas, Life Sciences). The amplification conditions were initial denaturation at 94°C for 3 min and 5 cycles at 94°C for 30 s, 56 to 46°C (-1°C each cycle), 72°C for 1 min followed by 35 cycles at 94°C for 30 s, 46°C for 1 min, 72°C for 1 min, and ends up with a final extension at 72°C for 7 min. PCR products were resolved on 3% agarose gel in TBE buffer at 80 V and the image was captured in a gel documentation system (Syngene, United Kingdom).

### Statistical Analysis

Analysis of variance for yield at each growing environment (Kanpur-stress, Kanpur-nonstress, Vamban-stress, Vamban-non-stress) was performed using a statistical package augmented RCBD (Aravind et al., 2020) in RStudio application using R (Programming Language for Statistical Analysis) (R Core Team, 2019). The yield data across four growing environments were graphically analyzed for interpreting G × E interaction using the “GGEBiplotGUI” statistical package in RStudio software using R (Frutos et al., 2014). GGE biplot methodology, which is composed of two concepts, the biplot concept and the GGE concept, was used for yield analysis across locations (Gabriel, 1971; Yan, 2001). This methodology uses a biplot to show the factors (G and G × E) that are important for evaluation of genotypes and that are also the sources of variation in G × E interaction analysis of multi- location trial data (Yan et al., 2000; Yan, 2001). All the physiological and biochemical data points were subjected to statistical analysis using Microsoft Excel software. For molecular data, all gels were scored manually, and data were input into Excel (Microsoft) spreadsheets. The band data were scored as a 1/0 (presence/absence) matrix. Genetic similarity coefficients of pair-wise comparisons among the accessions analyzed were calculated based on Jaccard’s similarity coefficient (Jaccard, 1908) within the Similarity for Qualitative Data (SIMQUAL) module of NTSYS 2.02i (Rohlf, 1998). The Unweighted Pair Group Method with Arithmetic Mean (UPGMA) clustering method was used to construct the dendrogram.

## Results

### Characterization of Heat Stress Conditions and Identification of Heat Tolerant Genotypes

**FIGURE 1:**
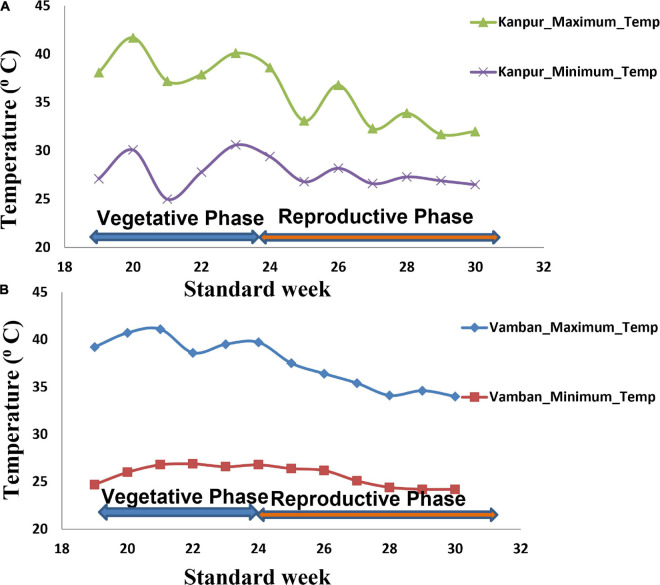
Temperature regime at two field trial sites during crop growth period (Kanpur and Vamban). **(A)** Temperature regime at the Kanpur location. **(B)** Temperature regime at the Vamban location.

**FIGURE 2:**
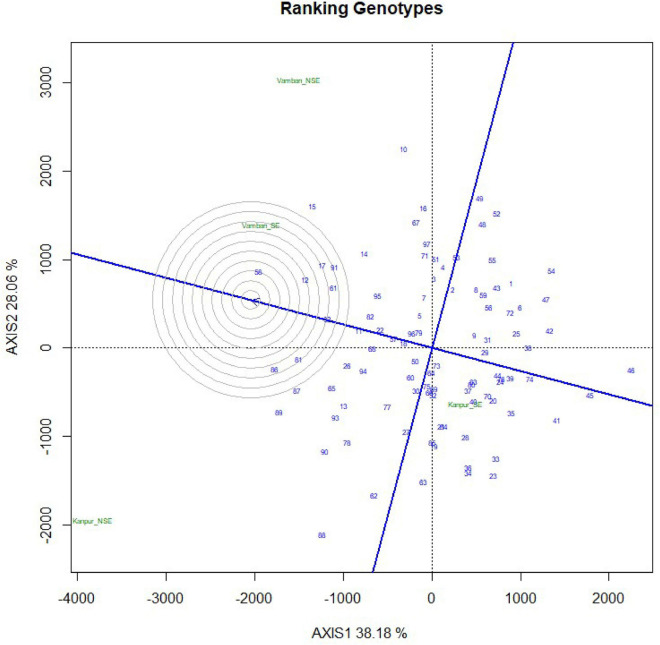
Ranking of 97 urdbean geneotypes by GGEBiplot analysis on the basis of yield data across four growing environments.

In the present study, natural heat stress conditions were determined based on mean yield obtained over 97 genotypes at two different locations (Kanpur and Vamban). The Kanpur location is situated in the northern part of India (26.28°N and 80.21°E) where early sown (mid-May) genotypes experienced heat stress with a rise of temperature (>40°C) coinciding with the reproductive stage, whereas late sown genotypes received moderate temperature (<40°C) during onset of flower followed by pod setting to grain development (Figure 1). Mean yield of early sown trials at this location was low (910 kg/ha) compared to late sown trial (1224 kg/ha) (*p* < 0.05). Similarly, the Vamban location is the extreme southern part of India (10.20°N, 78.50°E) where early sown crops are usually subjected to stress conditions with a rise of temperature to the extent of about 40°C during the reproductive stage (Figure 1). Early sown trials of this location showed low average yield (895 kg/ha) compared to late sown trials that had low temperature (<40°C) during the reproductive stage and higher mean yield (925 kg/ha) for 97 genotypes (Table 1) (*p* < 0.05). Analysis of variation over 97 genotypes for yield showed significant genotypic differences at *p* < 0.05 and < 0.01 probabilities under stress (early) and non-stress (late) conditions at both locations (Table 2). The heat sensitive and tolerant genotypes were identified at a preliminary stage based on the heat susceptibility index (HSI) under a field trial conducted in two contrasting environments. Sensitive genotypes were characterized with an HSI ranging from 0.08 to 3.19 at the Kanpur location and from 0.37 to 13.75 at the Vambam location, while HSI varied from -0.01 to -20.48 at Kanpur and -0.03 to –62.29 at Vambam among tolerant genotypes (Table 3). GGE biplot analysis identified most stable genotypes over the locations for yield (Figure 2).

**TABLE 1:** Yield of 97 urdbean genotypes grown in IIPR, Kanpur and TNAU, Vamban.

Sl. No.	Genotype	IIPR, Kanpur		TNAU, Vamban	
			
		SE^#^	NSE^#^	SE	NSE
1	IPU 91-7	862	598	858	1000
2	IPU 94-2	1016	998	1184	831
3	IPU 95-13	589	998	1011	1165
4	Pant U-30	516	1032	1302	725
5	LBG 20	529	1232	1030	876
6	UPU 97-10	429	732	929	471
7	NO 7668-4B	649	1132	1052	1020
8	PGRU 95018	722	865	1043	817
9	PGRU 95014	1036	1032	1002	595
10	PGRU 95016	1109	832	1787	1434
11	TU 99-293	756	1565	1086	1109
12	Pant U-19S	1056	1698	1445	1408
13	TU 99-2	1242	1832	859	1079
14	TU 91-22	902	1365	1506	1133
15	PLU-28	849	1498	1783	1401
16	PLU-1	1236	898	1541	1232
17	UH -177	1216	1498	1325	1683
18	BG-369	1400	1187	618	1702
19	BGP 21-28	1307	1420	388	954
20	U-9	780	1020	421	758
21	IC 106088	1700	1320	511	1023
22	UH 32-3	1427	1487	1254	959
23	STY 2868	1607	1187	178	773
24	UH 85-15	1220	920	537	847
25	IPU 90-32	1480	720	795	849
26	IPU 90-321	674	1720	947	1063
27	IPU 99-79	960	1520	421	1067
28	PLU-8	1140	1287	451	675
29	IPU 99-123	914	987	792	679
30	UH 80-26	1040	1387	661	1015
31	IPU 99-23	900	953	871	642
32	IC -21001	914	1253	447	1173
33	IPU 99-95	807	1187	245	518
34	IPU 99-40	1060	1387	325	526
35	PKGU-1	560	987	350	567
36	IPU 99-89	614	1353	217	587
37	NG-2119	860	1053	360	1119
38	NO- 5731	1347	765	838	537
39	Mash 1-1	880	965	722	396
40	UG 414	1454	1165	684	683
41	IPU 96-6	954	798	379	324
42	IPU 98/36	700	632	800	385
43	U 3108	1174	665	879	1130
44	DUS 34	540	932	472	781
45	STY 2289	1260	532	396	419
46	IC-65511	1047	165	171	677
47	UH 99-144	1607	432	790	1016
48	UH 86-5	520	632	1207	1014
49	STY-2834	680	498	1162	1383
50	PLU-429	860	1232	558	1377
51	PLU-144	574	965	1167	1076
52	UPU 85-86	1100	532	1335	1020
53	STY 2115	1134	765	1026	1388
54	IPU 99-31	914	398	939	760
55	U-132	760	632	967	1109
56	N0 7368-15	587	765	709	1067
57	UH 80-38	890	1343	851	1248
58	PLU-65	477	1876	1372	1680
59	BGP-247	1130	876	1162	672
60	PLU 456	1190	1343	666	1208
61	UG -218	870	1576	1332	1285
62	PDU-3	2210	1976	746	627
63	PLU -328	917	1676	444	476
64	NHKD-31	950	1276	726	938
65	STY-2824	677	1876	928	998
66	IPU 96-1	744	1309	538	1015
67	IPU2K-21	850	1176	1912	426
68	IPU-722	784	1476	820	1311
69	IC-10703	850	1276	564	1012
70	IPU 96-12	844	1076	559	625
71	IPU 99-22	424	1143	1487	609
72	PLU-703	510	809	975	441
73	PLU-557	1190	1343	1064	513
74	UG-378	224	876	583	268
75	IPU 99-128	830	1343	672	894
76	H-1	624	909	429	824
77	IPU 99-40	760	1542	513	1189
78	UPU 83-3	333	1975	672	716
79	PLU-662	647	1142	645	1393
80	UH 87-7	387	1208	745	370
81	UH 84-4	713	1908	966	1399
82	IPU 99-43	787	1475	1146	1075
83	PDU-1	1093	1208	935	323
84	IPU 99-18	1380	1342	526	867
85	IPU 99-16	1293	1475	538	743
86	IPU 99-200	733	1942	702	1888
87	UH 85-3	633	2142	1169	786
88	HPU-120	1013	2342	444	663
89	JU 78-27	833	2208	947	1130
90	PU-19	493	2075	615	918
91	IPU 99-209	453	1542	1388	1224
92	IPU 99-232	867	1675	1166	1317
93	IPU 99-221	820	2008	990	665
94	IPU 99-179	820	1775	1222	533
95	Uttara	998	1348	1173	1246
96	Shekhar-2	871	1282	943	1030
97	IPU 02-43	944	964	1294	1186
	**Mean**	**910**	**1224**	**859**	**925**
	**Standard error**	**34**	**45**	**38**	**35**

*^#^SE denotes stressed environment, NSE denotes non-stressed environment.*

**TABLE 2:** ANOVA of yield over stress and non-stress environments in IIPR, Kanpur and TNAU, Vamban.

Source	MS (Kanpur SE)	MS (Kanpur NSE)	MS (Vamban SE)	MS (Kanpur NSE)
Block (ignoring treatments)	342770**	685647**	143012**	207617**
Treatment (eliminating blocks)	100158**	111906*	121168**	115039**
Treatment: Check	20366	211098*	61912**	159219**
Treatment: Test and test vs. check	101754**	109922*	122365**	114146**
Residuals	11342	35865	1400	20663

* and ** denotes that mean square was significantly different at *p* < 0.05, *p* < 0.01, respectively.

**TABLE 3:** Heat susceptibility index (HSI) of 97 urdbean genotypes under IIPR, Kanpur and TNAU, Vamban condition.

Rank	IIPR, Kanpur (26.28°N and 80.21°E)		TNAU, Vamban (10.20°N, 78.50° E)	
		
	Genotype	HSI	Genotype	HSI
1	IC-65511	–20.48	IPU2K-21	–62.29
2	UH 99-144	–10.42	PDU-1	–33.83
3	STY 2289	–5.24	IPU 99-22	–25.74
4	IPU 99-31	–4.97	IPU 99-179	–23.08
5	UPU 85-86	–4.09	PLU-703	–21.62
6	IPU 90-32	–4.04	UG-378	–20.99
7	U 3108	–2.93	IPU 98/36	–19.25
8	NO- 5731	–2.91	PLU-557	–19.18
9	STY 2115	–1.85	UH 87-7	–18.1
10	IPU 91-7	–1.69	UPU 97-10	–17.36
11	PLU-1	–1.44	Mash 1-1	–14.7
12	STY-2834	–1.4	Pant U-30	–14.21
13	STY 2868	–1.36	BGP-247	–13.02
14	PGRU 95016	–1.28	PGRU 95014	–12.21
15	UH 85-15	–1.25	NO- 5731	–10.01
16	BGP-247	–1.11	IPU 99-221	–8.73
17	IC 106088	–1.1	UH 85-3	–8.7
18	UG 414	–0.95	IPU 94-2	–7.59
19	U-132	–0.78	IPU 99-23	–6.37
20	IPU 96-6	–0.75	TU 91-22	–5.88
21	BG-369	–0.69	UPU 85-86	–5.51
22	PDU-3	–0.45	UH 32-3	–5.49
23	IPU 98/36	–0.41	PGRU 95018	–4.94
24	IPU 99-18	–0.11	PLU-28	–4.87
25	IPU 94-2	–0.07	PLU-1	–4.48
26	PGRU 95014	–0.01	PGRU 95016	–4.4
27	IPU 02-43	0.08	IPU 99-31	–4.21
28	UH 32-3	0.15	UH 86-5	–3.4
29	IPU 99-23	0.21	PDU-3	–3.39
30	IPU 99-123	0.28	LBG 20	–3.14
31	BGP 21-28	0.3	IPU 96-6	–3.03
32	Mash 1-1	0.34	IPU 99-123	–2.97
33	PDU-1	0.36	IPU 99-209	–2.39
34	PLU 456	0.44	IPU 02-43	–1.63
35	PLU-557	0.44	PLU-144	–1.51
36	PLU-8	0.44	IPU 99-43	–1.18
37	IPU 99-16	0.47	UG -218	–0.65
38	PGRU 95018	0.63	NO 7668-4B	–0.56
39	UH 86-5	0.68	Pant U-19S	–0.47
40	NG-2119	0.7	UG 414	–0.03
41	UH -177	0.72	TU 99-293	0.37
42	IPU 96-12	0.83	STY 2289	0.98
43	N0 7368-15	0.89	Uttara	1.05
44	U-9	0.9	UPU 83-3	1.1
45	IPU 99-40	0.9	IPU 90-32	1.14
46	UH 80-26	0.96	PLU -328	1.2
47	NHKD-31	0.98	STY-2824	1.25
48	Uttara	0.99	Shekhar-2	1.51
49	IC -21001	1.04	IPU 96-12	1.89
50	IPU2K-21	1.06	IPU 90-321	1.95
51	PLU-429	1.16	IPU 99-232	2.05
52	H-1	1.2	U-132	2.29
53	IPU 99-95	1.23	IPU 95-13	2.36
54	Shekhar-2	1.23	IPU 91-7	2.54
55	TU 99-2	1.23	STY-2834	2.85
56	IC-10703	1.28	JU 78-27	2.89
57	UH 80-38	1.29	PLU-65	3.27
58	TU 91-22	1.3	TU 99-2	3.64
59	IPU 99-79	1.41	UH -177	3.8
60	PLU-703	1.42	U 3108	3.97
61	Pant U-19S	1.45	UH 99-144	3.97
62	IPU 99-128	1.46	NHKD-31	4.04
63	PLU-144	1.55	IPU 99-128	4.43
64	IPU 95-13	1.57	STY 2115	4.66
65	UPU 97-10	1.59	IPU 99-16	4.93
66	DUS 34	1.61	UH 84-4	5.53
67	NO 7668-4B	1.63	UH 80-38	5.68
68	IPU 96-1	1.65	PU-19	5.89
69	PKGU-1	1.66	HPU-120	5.9
70	PLU-28	1.66	PLU-8	5.93
71	PLU-662	1.66	N0 7368-15	5.99
72	UG -218	1.72	UH 80-26	6.23
73	PLU -328	1.74	UH 85-15	6.54
74	IPU 99-43	1.79	IPU-722	6.69
75	IPU-722	1.8	IPU 99-40	6.82
76	IPU 99-232	1.85	PKGU-1	6.83
77	Pant U-30	1.92	IPU 99-18	7.02
78	IPU 99-40	1.94	DUS 34	7.07
79	TU 99-293	1.98	IC-10703	7.91
80	IPU 99-179	2.06	U-9	7.94
81	IPU 99-89	2.09	PLU 456	8.01
82	HPU-120	2.17	IPU 96-1	8.39
83	LBG 20	2.19	H-1	8.56
84	IPU 99-221	2.27	IC 106088	8.94
85	IPU 90-321	2.33	IPU 99-95	9.41
86	IPU 99-200	2.39	PLU-662	9.59
87	JU 78-27	2.39	IPU 99-40	10.15
88	UH 84-4	2.4	BGP 21-28	10.59
89	IPU 99-22	2.41	PLU-429	10.62
90	STY-2824	2.45	IPU 99-79	10.81
91	UH 87-7	2.6	IC -21001	11.05
92	UH 85-3	2.7	IPU 99-200	11.22
93	IPU 99-209	2.71	IPU 99-89	11.26
94	UG-378	2.85	BG-369	11.37
95	PLU-65	2.86	NG-2119	12.11
96	PU-19	2.92	IC-65511	13.35
97	UPU 83-3	3.19	STY 2868	13.75

### Physiological Characterization

Field trials identified 8 highly heat tolerant and 35 highly heat sensitive genotypes based on HSI (Table 4). Among these, six highly sensitive (IPU 99-200, IC 21001, Shekhar 2, PU 19, H-1, PKGU 1) and seven highly tolerant (UPU 85-86, IPU 94-2, IPU 98/36, NO- 5731, PGRU 95016, PLU 1, BGP 247) genotypes, showing stable HSI over both locations, were used for further physiological analyses (Table 4). These genotypes were grown under a controlled environment right from seedling stage until maturity in a high thermal regime (40/25°C: maximum/minimum) with high humidity and under optimum irrigation and soil fertility.

**TABLE 4:** Heat-tolerant and heat-sensitive urdbean genotypes over both the locations (IIPR, Kanpur and TNAU, Vamban) based on HSI.

Sl. No.	Genotypes^#^	HSI at IIPR, Kanpur	HSI at TNAU, Vamban

	*Heat tolerant*		
1	**UPU 85-86**	–4.09	–5.51
2	**IPU 94-2**	–0.07	–7.59
3	**IPU 98/36**	–0.41	–19.25
4	**NO- 5731**	–2.91	–10.01
5	**PGRU 95014**	–0.01	–12.21
6	**PGRU 95016**	–1.28	–4.40
7	**PLU-1**	–1.44	–4.48
8	**BGP-247**	–1.11	–13.02
	***Heat sensitive***		
1	DUS 34	1.61	7.07
2	**H-1**	1.20	8.56
3	**HPU-120**	2.17	5.90
4	**IC -21001**	1.04	11.05
5	IC-10703	1.28	7.91
6	IPU 90-321	2.33	1.95
7	IPU 95-13	1.57	2.36
8	IPU 96-1	1.65	8.39
9	IPU 96-12	0.83	1.89
10	IPU 99-128	1.46	4.43
11	**IPU 99-200**	2.39	11.22
12	IPU 99-232	1.85	2.05
13	IPU 99-40	1.94	6.82
14	IPU 99-79	1.41	10.81
15	IPU 99-89	2.09	11.26
16	IPU 99-95	1.23	9.41
17	IPU-722	1.80	6.69
18	JU 78-27	2.39	2.89
19	NO 7368-15	0.89	5.99
20	NHKD-31	0.98	4.04
21	**PKGU-1**	1.66	6.83
22	PLU -328	1.74	1.20
23	PLU-429	1.16	10.62
24	PLU-65	2.86	3.27
25	PLU-662	1.66	9.59
26	**Shekhar-2**	1.23	1.51
27	STY-2824	2.45	1.25
28	TU 99-2	1.23	3.64
29	U-9	0.90	7.94
30	UH -177	0.72	3.80
31	UH 80-26	0.96	6.23
32	UH 84-4	2.40	5.53
33	UPU 83-3	3.19	1.10
34	**Uttara**	0.99	1.05
35	**PU-19**	2.92	5.89

*^#^ Genotypes in bold font were used for physiological, biochemical, and molecular characterization.*

### Physiological Status Under Stress Environment

Changes in the physiological status were observed between two contrasting groups having different degrees of heat sensitivity when they were grown under higher thermal regime (40/25°C). The genotypic variability in nitrogen balance index (NBI) and chlorophyll (Chl) was significant (*p* < 0.05). No significant differences were observed among tested genotypes for anthocyanin and flavanol content when subjected to heat stress (Table 5). However, group comparison (heat tolerant vs. heat sensitive) (*t*-test) revealed significant differences in leaf nitrogen status (NBI) and anthocyanin (Anth) at *p* < 0.01 (Table 6).

**TABLE 5:** Analysis of variance of fluorescence parameters of 13 tested urdbean genotypes.

		Means of square				
			
Source	Degrees of freedom	Leaf nitrogen balance index	Chlorophyll	Flavanol	Anthocyanin	Membrane stability
Genotypes	12	295**	31**	0.003	0.002	452.9**
Error	14	75	5	0.002	0.001	58.10
Total	26	360	36	0.005	0.003	511

** Significant at *p* < 0.01.

**TABLE 6:** Leaf nitrogen balance index (NBI), chlorophyll (SPAD), epidermal flavanols, and anthocyanins in different urdbean genotypes grown under 42/30°C max/min temperature.

Genotype	NBI	Chlorophyll	Flavanol	Anthocyanin
*Heat tolerant genotypes*				
UPU 85-86	64.6 ± 1.25	21.1 ± 0.05	0.3 ± 0.01	0.00 ± 0.00
IPU 94-2	49.7 ± 6.70	17.2 ± 2.75	0.3 ± 0.01	0.00 ± 0.00
IPU 98/36	36.9 ± 5.60	9.7 ± 1.30	0.3 ± 0.08	0.03 ± 0.02
NO- 5731	49.4 ± 7.05	12.7 ± 0.45	0.2 ± 0.02	0.00 ± 0.00
PGRU 95016	56.8 ± 0.25	16.8 ± 1.15	0.3 ± 0.04	0.00 ± 0.00
PLU 1	56.1 ± 1.90	15.3 ± 0.30	0.3 ± 0.00	0.00 ± 0.00
BGP 247	61.9 ± 1.90	18.0 ± 0.00	0.3 ± 0.01	0.00 ± 0.00
**Range**	**36.9-64.6**	**9.7-21.1**	**0.2-0.3**	**0.00-0.00**
***Heat sensitive genotypes***				
IPU 99-200	48.7 ± 2.75	19.4 ± 0.85	0.4 ± 0.01	0.00 ± 0.01
IC 21001	51.7 ± 11.70	15.6 ± 3.35	0.3 ± 0.00	0.02 ± 0.02
Shekhar 2	37.3 ± 15.65	13.4 ± 2.65	0.3 ± 0.03	0.06 ± 0.06
PU 19	30.3 ± 5.30	13.1 ± 2.65	0.3 ± 0.06	0.08 ± 0.01
H-1	43.7 ± 3.60	13.9 ± 1.50	0.3 ± 0.01	0.01 ± 0.01
PKGU 1	23.0 ± 0.70	6.2 ± 0.25	0.3 ± 0.00	0.09 ± 0.00
**Range**	**23.0-48.7**	**6.2-19.4**	**0.3-0.4**	**0.00-0.09**
C.D. (5%)	18.52	4.82	N/A	N/A
SE(m)	6.13	1.59	0.03	0.02
SE(d)	8.66	2.25	0.04	0.03
C.V.	19.07	15.76	12.72	133.77
***t-Value***	**2.57**	**−**	**−**	**-3.045**
***p-Value (p < 0.01)***	**0.0129**	**−**	**−**	**0.0062**

In the present study, a range of genetic variability was observed higher among seven heat tolerant genotypes (36.9-64.6 and 9.7-21.1) compared to six heat sensitive genotypes (23.0-48.7 and 6.2-19.4) for NBI and chlorophyll content, respectively (Table 6).

### Membrane Stability

Analysis of variance showed significant differences among the studied urdbean genotypes for membrane stability index (Table 5). It ranged from 32.3% to 74.5% in sensitive genotypes while it ranged from 34.5 to 62.8% in tolerant genotypes (Table 7). Although membrane stability was observed significantly higher in the sensitive genotype IPU 99-200 (74.5%), membrane stability was on average higher among tolerant genotypes compared with sensitive genotypes (Table 7). Among tolerant genotypes, maximum membrane stability was observed in PLU-1 (62.8 %) followed by UPU 85-86 (60.7 %) (Table 7).

**TABLE 7:** Membrane stability of 13 urdbean genotypes.

Genotype	Membrane stability (%)
*Heat sensitive genotypes*	
IPU 99-200	74.5 ± 4.3
IC 21001	44.3 ± 3.7
H-1	55.3 ± 3.9
PKGU-1	32.3 ± 3.5
Shekhar 2	38.4 ± 3.4
PU 19	42.3 ± 4.5
***Heat tolerant genotypes***	
UPU 85-86	60.70 ± 5.4
IPU 94-2	34.50 ± 4.6
IPU 98/36	49.60 ± 7.4
NO- 5731	42.90 ± 4.2
PGRU 95016	56.40 ± 3.9
PLU-1	62.80 ± 3.7
BGP-247	55.80 ± 4.8

### Correlation Analysis Among Nitrogen Balance Index, Chlorophyll, Flavanol, Anthocyanin Contents, and Membrane Stability

A highly significant (*p* < 0.01) positive correlation (*r*^2^ = 0.85) was observed between NBI and chlorophyll content. Also, correlation of anthocyanin content with chlorophyll content (*r*^2^ = −0.72) and NBI (*r*^2^ = −0.89) was highly significant (*p* < 0.01) and negative in nature (Table 8).

**TABLE 8:** Correlation analysis of nitrogen balance index (NBI), chlorophyll, flavanol, anthocyanin contents, and membrane stability.

	NBI	Chlorophyll	Flavanol	Anthocyanin
**NBI**				
**Chlorophyll**	0.85**			
**Flavanol**	–0.01	0.34		
**Anthocyanin**	−0.89**	−0.72**	−8.051E-17	
**Membrane stability**	0.02	0.04	3.655E-02	0.18

**Significant at *p* < 0.01.

### Photosynthetic Electron Transport Rate

**FIGURE 3:**
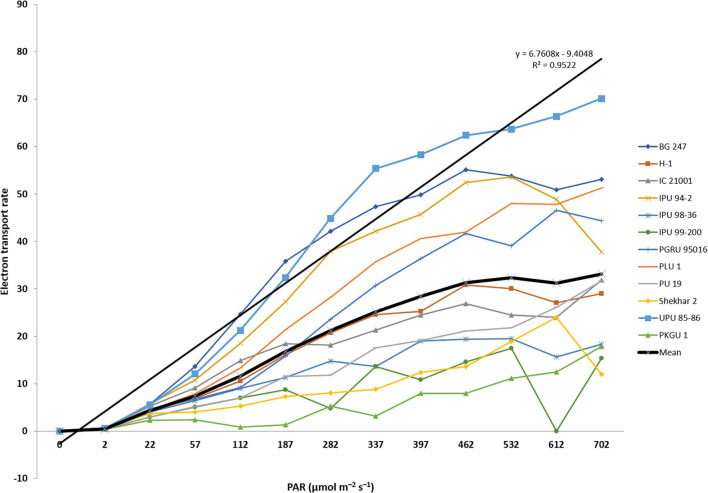
Electron transport rate (ETR) of heat tolerant and sensitive urdbean genotypes over increased irradiation (PAR).

Photosynthetic electron transport rate (ETR) was analyzed among 13 heat sensitive and tolerant urdbean genotypes at increasing levels of PAR (photosynthetically active radiation). The analysis of variance showed significant differences among genotypes for ETR at different levels of PAR irradiances (Table 9). These differences were more noticeable with progressive increase in the levels of PAR irradiances among test genotypes (Figure 3). The interaction of PAR irradiances with genotypes (PAR × genotypes) was also observed to be significant (Table 9). In the present study, higher levels of irradiances were found to be the main determinant of differentiating thermotolerance based on photosynthetic performance in all studied genotypes after heat shock (43°C for 1 h). The light-saturated ETR was obtained almost in all test genotypes within the PAR ranging from 400 to 500 μmol m^–2^ s^–1^ (Figure 3). The PAR irradiances exceeding the saturation range of 400-500 μmol m^–2^ s^–1^ pose damaging effects on photosynthetic systems due to excessive production of superoxide radicals. Perhaps tolerant genotypes exposed to combined stress of high PAR irradiances and heat shock that still maintain high ETR have alternate mechanisms scavenging harmful radicals. However, under light limiting conditions below 400-500 μmol m^–2^ s^–1^, genotype performances were assessed primarily under single stress that was only heat shock. Therefore, the ability of heat tolerance can be detected but cannot be truly assessed under light limiting conditions. Realizing the facts under field conditions, actual heat stress is often combined or integrated with high solar radiation and the crop is forced to experience the combined effects of heat and high light stress, and assimilate production is virtually collapsed resulting in massive yield loss. In the present study, the light-saturated photosynthetic electron transport rate was observed higher than the mean of all test genotypes in most of the tolerant genotypes. Five out of seven heat tolerant genotypes (UPU 85-86, BG 247, PLU 1, PGRU 95016, and IPU 94-2) showed higher photosynthetic ETR than the rest of the tested urdbean genotypes.

**TABLE 9:** Analysis of variance for electron transport rate.

Source	Degrees of freedom	Means of square
Genotypes	12	0.766**
PAR	12	1.746**
Genotypes × PAR	144	0.013**
Error	676	0.003
Total	844	

**Significant at *p* < 0.01.

### Fluorescence Parameters in Highly Heat Tolerant and Sensitive Genotypes

**FIGURE 4:**
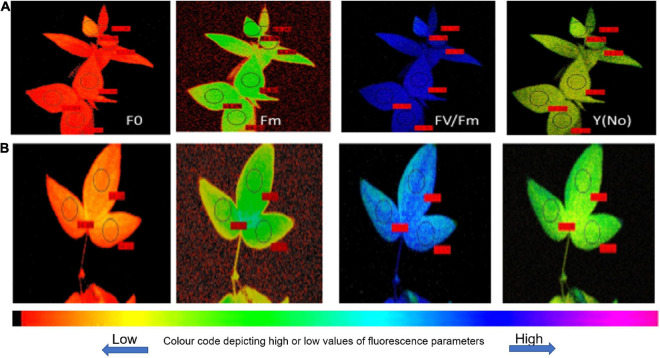
Fluorescence images for heat-tolerant **(A)** (UPU85-86) and heat-sensitive genotypes **(B)** (PKGU-1).

Further studies remained confined to two extreme genotypes having a high degree of heat tolerance (UPU 85-86) and sensitivity (PKGU-1) based on the field trials and precision phenotyping. The different fluorescence parameters were analyzed to distinguish highly tolerant (UPU 85-86) and highly sensitive (PKGU-1) genotypes. Analysis of variance of fluorescence parameters between heat tolerant (UPU 85-86) and heat sensitive genotypes (PKGU-1) showed significant differences (Table 10). The mean value of these parameters is given in Table 11. The observed increase in average minimal fluorescence (F_0_) and corresponding decline in the maximal fluorescence (F_*m*_) and variable fluorescence (Fv) in preheated leaves of sensitive genotype PKGU-1 was the strong indicator of distortion of PS II. Consequently, reduction in the quantum yield of PS II was evident in the sensitive one as compared to the tolerant genotype. The decrease in the quantum yield with concomitant rise in the quantum yield of non-regulated energy dissipation [Y(NO) = 0.439] in the sensitive genotype compared to the tolerant genotype [Y(NO) = 0.253] suggested dissipation of absorbed light energy into wasteful thermal or fluorescence quenching, leading to reduction in the photosynthetic efficiency especially targeting the light reaction. While average maximal fluorescence, variable fluorescence, and quantum yield were higher in the tolerant genotype (Fm = 0.277, Fv = 0.215, and F_*v*_/F_*m*_ = 0.749, respectively) than the sensitive one (Fm = 0.257, Fv = 0.155, and F_*v*_/F_*m*_ = 0.544, respectively). Despite the differences in average values of these two genotypes, analysis of variance showed significant differences only for minimal fluorescence (F_0_), quantum yield (F_*v*_/F_*m*_), and quantum yield of non-regulated energy dissipation [Y(NO)] at *p* =< 0.01 (Table 10). The significant differences for quantum yield suggest that these two test genotypes responded differently under heat stress conditions as depicted in fluorescence images for heat tolerant (UPU 85-86) and heat sensitive genotypes (PKGU-1) (Figure 4 and Table 10).

**TABLE 10:** Analysis of variance of fluorescence parameters between heat-tolerant (UPU85-86) and heat-sensitive genotypes (PKGU-1).

		Means of square				
		
Source	Degrees of freedom	F_0_ (Minimal fluorescence)	F_*m*_ (Maximal fluorescence)	F_*v*_ (Variable fluorescence)	F_*v*_/F_*m*_ (Quantum yield)	Y(NO) (Quantum yield of non-regulated energy dissipation)
Genotypes	1	0.005**	0.001	0.011	0.126**	0.104**
Error	10	0	0.009	0.007	0.006	0.007
Total	11	0.005	0.1	0.018	0.132	0.111

**Significant at *p* < 0.01.

**TABLE 11:** Fluorescence parameters to differentiate two contrasting heat tolerant and sensitive urdbean genotypes grown at 42/25°C max/min temperature.

Treatment	Minimal fluorescence, F_0_	Maximal fluorescence, Fm	Variable fluorescence, Fv	Quantum yield, Fv/Fm	Quantum yield of non-regulated energy dissipation, Y(NO)
	Mean ± SE	Mean ± SE	Mean ± SE	Mean ± SE	Mean ± SE
UPU 85-86	0.062 ± 0.007	0.277 ± 0.036	0.215 ± 0.031	0.749 ± 0.010	0.253 ± 0.011
PKGU 1	0.102 ± 0.008	0.257 ± 0.043	0.155 ± 0.036	0.544 ± 0.045	0.439 ± 0.049
C.D.	0.024	N/A	N/A	0.105	0.113
SE(m)	0.008	0.039	0.034	0.033	0.035
SE(d)	0.011	0.056	0.048	0.047	0.050
C.V.	22.542	36.094	44.872	12.464	25.015

### Phenotyping Heat Tolerant and Sensitive Genotypes Using Chlorophyll Fluorescence Image-Based Diagnostics

The quantitative values of fluorescence parameters as shown in Table 11 were transformed into color fluorescence images and the differences in the image pattern between heat tolerant and sensitive genotypes could be easily distinguishable by different shades of color as indicated in the color code bar appended with Figure 4 having low or high values. For example, a deep blue color of quantum yield as shown in the heat tolerant genotype UPU 85-86 is attributed to high quantum yield of PS II, while similar treatment resulted in fading of the blue color to convert to sky blue, representing reduction in the quantum yield. In a similar manner, the heat sensitive genotype PKGU-1 had higher values of minimal fluorescence (F_0_) and quantum yield of non-regulated energy dissipation [Y(NO)], which has been well depicted by changes in the color of the corresponding fluorescence images largely differing from the heat tolerant genotype (UPU 85-86). The maximum fluorescence F_*m*_ also decreased compared to the heat tolerant ones, which could be easily defined by changes in the color of fluorescence images between these two categories.

### Auto-Recovery of Fluorescence Parameters During Light-Dark Transition

**FIGURE 5:**
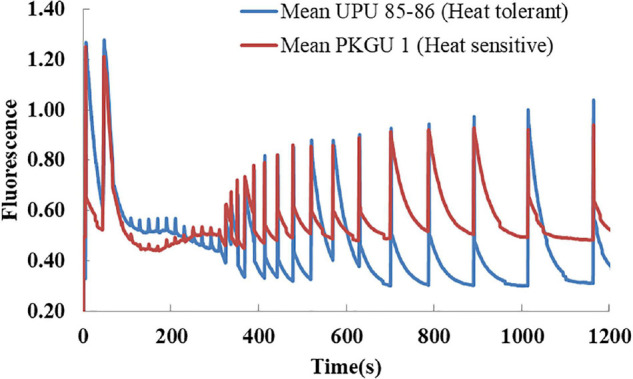
Relative auto-recovery of altered fluorescence during light to dark transition in two contrasting urdbean genotypes (heat-tolerant UPU85-86 and heat-sensitive PKGU-1).

The repeated flashes of saturated pulses were triggered at regular intervals upon leaves of heat tolerant (UPU 85-86) and sensitive genotype (PKGU-1) adapted to actinic light (200 μmol m^–2^ s^–1^) continuously for 300 s to obtain F_*v*_/F_*m*_, F_*m*_, and F_0_ after each saturation pulse. Thereafter, actinic light switched off to allow leaves for light to dark transitions to assess the recovery of F_*v*_/F_*m*_, F_*m*_, and F_0_ (Figure 5). The results showed that quantum yield (F_*v*_/F_*m*_) decreased drastically in the heat sensitive genotype (PKGU-1) when leaves were continuously exposed to actinic light and dark transition. The quantum yield (F_*v*_/F_*m*_) could not recover to the pre-illumination value of 0.50 (Figure 5). Notably minimal fluorescence F_0_ remained higher and unaltered during the entire dark period suggesting damage or distortion of PS II in sensitive genotypes. Whereas, quantum yield F_*v*_/F_*m*_ remained higher in the tolerant one (UPU 85-86) during light phase and completely and reversibly recovered to a pre-illumination value of 0.7 in the dark phase (Figure 5).

**FIGURE 6:**
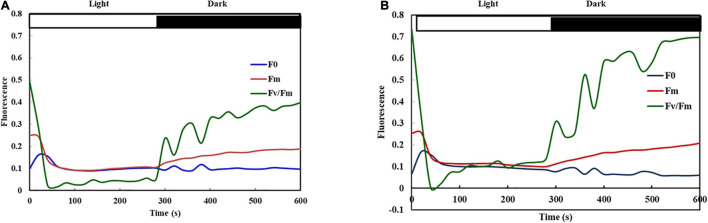
Fluorescence kinetics of heat sensitive (PKGU-1; **A**) and heat tolerant (UPU 85-86; **B**) urdbean genotypes during light to dark transition. The dark phase started from 350 s.

Fluorescence kinetics of high temperature grown heat sensitive (PKGU 1) and heat tolerant (UPU 85-86) genotypes were studied during light to dark transition (Figure 6). Maximum fluorescence (F_*m*_) peak was observed in both contrasting lines immediately after dark adaptation and thereafter the time course trend revealed faster quenching or declining of F_*m*_ in both heat sensitive and tolerant genotypes along with shorter peaks of F_*m*_ (Figure 6) throughout the period until leaves were exposed to light. At the beginning of the dark phase starting after 350 s, the F_*m*_ values started rising and the time taken to decrease in the F_*m*_ in these two contrasting genotypes could differentiate them on the basis of their differential sensitivity toward heat stress (Table 12).

**TABLE 12:** Half quenching time of Fm for heat tolerant (UPU85-86) and sensitive (PKGU-1) urdbean genotypes.

Dark phase starting point (s)	Half quenching time of Fm of heat tolerant genotype UPU 85-86 (s)	Half quenching time of Fm of heat sensitive genotype PKGU -1 (s)
400	0.0	0
420	0.0	0
440	0.0	0
520	0.0	0
580	0.0	0
700	0.0	50.0
800	0.0	50.0
900	0.0	50.0
1000	25.0	0.0
1200	0.0	0.0

### Biochemical Analysis of Heat Sensitive and Tolerant Genotypes

**FIGURE 7:**
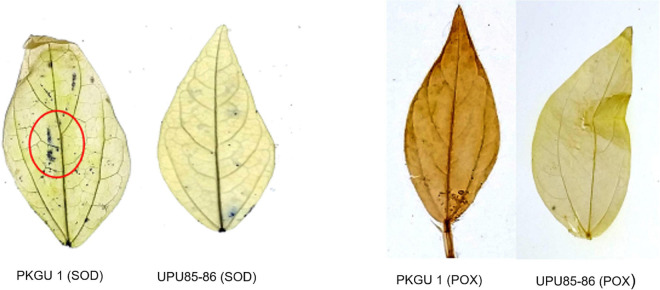
*In vivo* visualization of antioxidant activity for superoxide radicals and hydrogen peroxides in heat sensitive and tolerant genotypes.

Antioxidative enzymes such as superoxide dismutase (SOD) and peroxidase (POX) play important roles in protecting cellular systems like membranes, proteins, and enzymes by scavenging superoxide radicals and hydrogen peroxides produced during detrimental temperature beyond the threshold level of tolerance which is shown by *in vivo* visualization of superoxide radicals and hydrogen peroxides in Figure 7. High antioxidant activity confers tolerance to heat stress, which was represented by less blue color staining zones (formazan deposits) in the leaf (superoxide radicals) or lack of dark brown staining (hydrogen peroxides) as observed in the heat tolerant genotype UPU 85-86 (Figure 7). On the contrary, more intense blue crystal patches over leaf surfaces (superoxide radicals) and intense brown coloration (hydrogen peroxides) were the indicators of low antioxidative enzyme activities in heat sensitive genotypes (PKGU-1).

### Molecular Characterization

**FIGURE 8:**
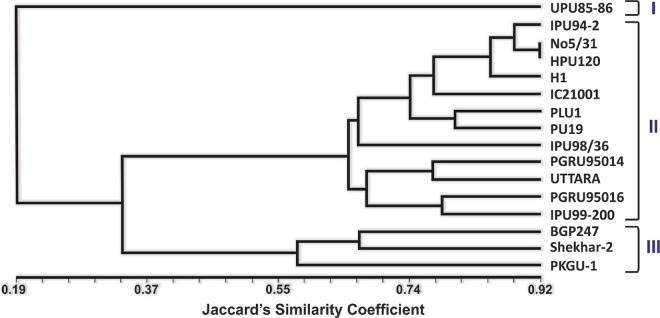
Unweighted Pair Group Method with Arithmetic Mean-based dendrogram showing the clustering of different urdbean genotypes.

**FIGURE 9:**
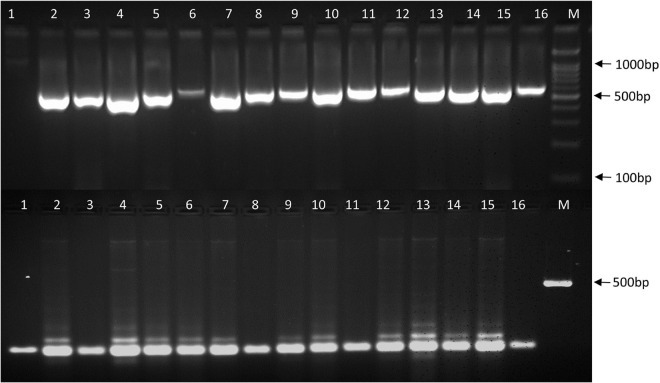
Amplification profiles of 16 urdbean genotypes using SSR primers (DR04 and YMVSSR74). The numbers represent the genotypes 1: UPU-85-86; 2: IPU94-2; 3: IPU-98/36; 4: NO. 5/31; 5: PGRU-95014; 6: PGRU-95016; 7: PLU-1, 8: BGP-247; 9: IPU99-200; 10: IC-21001; 11: SEKHAR-2; 12: UTTARA; 13: PU-19; 14: HPU-120; 15: H-1; 16: PKGU-1; M: DNA marker.

Twenty heat related polymorphic SSR markers were able to group the 16 urdbean genotypes into three major clusters as shown in Figure 8. The representative amplification profiles of the 16 urdbean genotypes using SSR markers are illustrated in Figure 9. Polymorphic information content ranged from 0.23 to 0.88 with an average value of 0.55 and one to three alleles were amplified by markers (Table 13). Cluster I is comprised of a single genotype UPU 85-86. Cluster II consisted of a mixture of six heat tolerant (IPU94-2, NO.5/31, PLU1, IPU98-36, PGRU-95014, PGRU-95016) and six heat sensitive genotypes (HPU120, H1, IC21001, PU19, UTTARA, IPU99-200). Cluster III housed two sensitive (SHEKHAR-2 and PKGU-1) and one heat tolerant genotype (BGP-247). The heat tolerant genotype UPU 85-86 and the heat sensitive genotype PKGU-1 were genetically distinct and were resolved at the extremes of the dendrogram. Thus, UPU 85-86 and PKGU-1 are genetically distinct as well as contrasting for heat tolerance.

**TABLE 13:** Details of genic-SSRs used for genotyping 16 urdbean genotypes.

SI. no.	Primer	Forward sequence Reverse sequence	Tm values	Product size (bp)	No. of alleles amplified	PIC	Function annotation	References
1	TWSSR14	CCGGAAAAGGGAAAACTACATT GCAGAACAGCAGAAACCTCTTT	56.5 58.4	300	1	0	*Vigna angularis var. angularis* DNA, chromosome 1	Raizada, 2020
2	TWSSR15	TCCTGTTCATCCTGATCTTCTTC TAACAAACCCCAAACACACAAC	58.9 56.5	100	1	0.44	Vacuolar sorting receptor	Raizada, 2020
3	TWSSR1	AGAGGGATGGGAGAGGGAT GAAGAAATTGGTGAGACCCAAA	58.8 56.5	180	1	0.61	Protein ABCI7	Raizada, 2020
4	TWSSR34	CGTGCTCGCAACTTCTCTC TCACCACTCTTCTTGTTGTGCT	58.8 58.4	600	1	0.44	60S ribosomal protein L29–1	Raizada, 2020
5	TWSSR4	AACCTTGTCGTGTTCAATCCTT CAAAGATCAGTGTTTCCCACAA	56.5 56.5	220	1	0.75	Transcription factor bHLH143-like	Raizada, 2020
6	TWSSR20	TCGTTAAGAAGGTCAAATGGGT GGCTCGATTGATGAAGAAGGT	56.5 57.9	170	1	0.23	Transcription factor 25	Raizada, 2020
7	TWSSR24	AGTGTTTTGGATTATGGATGGG TCACCAGTTTTATGCACCAGAG	56.5 58.4	200	1	0.61	Glycerol-3-phosphate transporter 4	Raizada, 2020
8	TWSSR72	GGAAAGAGCAGACCTTGACATC CCCAACAAAGCACAGAAACAA	60.3 55.9	280	1	0.23	*Vigna angularis* uncharacterized LOC108326918	Raizada, 2020
9	TWSSR12	GAACTGTATGTAGCAGGGGCTC AGAGGAGACAAAACGCAGAGAT	62.1 58.4	280-300	2	0.61	*Vigna radiata* var. *radiata* uncharacterized LOC106765753	Raizada, 2020
10	YMVSSR74	GAGAGTTTGAGGAGCGGTTG TTGACCTCGTGCAAGCATAG	59.3 57.3	200-220	2	0.53	*Glycine max* heat shock protein (SB100)	Raizada, 2020
11	D102666	TACGAGGCATTTGGTTTGACAGTG AGCCGGTTCCTCCATTTCTT	61 57.3	500-600	2	0.75	*Vigna radiata* sucrose synthase	Venkatesha et al., 2007
12	AF077224	AGCTGAAGCCGCCACCATA AGCAGCAGCCTTAAACTCATCAA	58.8 58.9	700-800	3	0.88	*Glycine max* Fe-super oxide dismutase	Venkatesha et al., 2007
13	AB056453	CCCTCGGCTATAGCATTGAAGAC ACGCATAAACAAAGAGGCTGGACT	62.4 61	600-700	2	0.76	*Vigna unguiculata* S-adenosylmethionine decarboxylase	Venkatesha et al., 2007
14	CA906101	AACACGCGGTACTACGAAATCCTC CTCCGCGTCTCTGTCTCCTACCTC	62.7 67.8	400-600	3	0.86	*Vigna radiata* var. *radiata* dnaJ Protein	Venkatesha et al., 2007
15	CLM446	TCCTCTGTCCTTTCTTTCTCTTT TGGAAGTTAAGACCCACCAG	57.1 57.3	200	1	0.61	*Vigna unguiculata* alpha/beta hydrolase domain-containing protein	Xu et al., 2011
16	X91836-5C	CCGGAAACATGGCATTATTATTAG CCATTGCCTCGTTCCCATCTT	57.6 59.8	500	1	0.44	*Vigna unguiculata* extension 2 like	Gowda, 2008
17	CLM438	TAAAGCCTCCACCCTTCTTT TTCCATGAGTCACCCACTTT	55.3 55.3	200	1	0.34	myb-related transcription factor [*Arabidopsis thaliana*]	Xu et al., 2011
18	CLM443	GGATGCGTCTAAGCCTGTTA CACATGACGAAAGAGATGGA	57.3 55.3	300-400	3	0.79	Putative serine acetyltransferase [*Oryza sativa* (japonica cultivar-group)]	Xu et al., 2011
19	CLM447	GGAAACATGACCTTGACGTT GACAGATGCGTGTGTCCATA	55.3 57.3	250	1	0.33	Putative nuclear ribonuclease Z [*Oryza sativa* (japonica cultivar-group)]	Xu et al., 2011
20	CLM451	ACAATGGACACAACCAACCT CTTGAAGACAGGTTCCTGAAA	55.3 55.9	300	1	0.44	Leaf senescence-associated receptor-like protein kinase (*Phaseolus vulgaris*)	Xu et al., 2011
21	CLM1000	GAGTCTATCGCTTTCTCAGTC CAGTAGGAACCCTCTTGATTT	57.9 55.9	300	1	0.44	Putative uncharacterized protein	Xu et al., 2011

## Discussion

In the present study, a panel of 97 urdbean genotypes was assessed under heat stress and non-heat stress conditions at two field locations. Stress conditions of a location have been decided based on average yield of trials and high temperature during early sown trials compared to lower temperature during late sown trials in the present study. The significant genotypic differences among tested urdbean genotypes for yield indicated the availability of heat tolerant genotypes. In other *Vigna* species, genetic variability for yield and yield contributing traits have also been observed under heat stress conditions (Basu et al., 2019). Heat susceptibility index (HSI) based on yield potential of a particular genotype under heat stress and non-stress conditions helped to distinguish heat sensitive and tolerant genotypes. This led to the identification of 8 highly heat tolerant and 35 highly heat sensitive genotypes. In the present study, tolerant genotypes had negative HSI due to higher yield under stress conditions compared to non-stress conditions, while highly sensitive genotypes had positive high HSI at both locations. HSI is a widely used method for identification of heat tolerant genotypes and has been used to identify heat tolerant genotypes in other crops (Pandey et al., 2015; Bhandari et al., 2017; Sita et al., 2017). Further, GGE biplot analysis identified most stable genotypes over the locations for yield.

The leaf NBI and chlorophyll content based on SPAD value showed significant differences among genotypes and both these parameters were higher in seven heat tolerant genotypes (36.9-64.6 and 9.7-21.1) compared to six heat sensitive genotypes (23.0-48.7 and 6.2-19.4). These results indicated enhanced chlorophyll synthesis and thereby maintaining higher leaf nitrogen balance in the heat tolerant genotype when grown at high temperature. The decrease in chlorophyll content in the heat sensitive genotypes reduced photosynthetic capacity and induced faster senescence due to high temperature as reported earlier in wheat and cucumber (Tewari and Tripathy, 1998). In the present study, no significant differences were observed among the heat sensitive and tolerant genotypes for leaf anthocyanin pigment. However, significantly higher leaf anthocyanin content (*p* < 0.01) was found in heat sensitive genotypes indicating the role of anthocyanin pigment for protecting the survival of sensitive genotypes from high temperature stress (Table 6). In other crops, the role of anthocyanin pigment accumulation has also been shown in response to various abiotic stresses (Castellarin et al., 2007) due to its antioxidant properties and photoprotection ability (Abdel-Aal et al., 2008).

Membrane stability index (MSI) under heat stress is one of the important physiological traits for identification of heat tolerant genotypes (Sikder et al., 2001; Dhanda and Munjal, 2006; Ashraf and Foolad, 2007) because high temperature affects several physiological processes such as photosynthesis and respiration through conformational changes in cell membrane bound proteins (Blum et al., 2001). In cowpea and *Brassica*, this trait has been used to identify potential heat tolerant genotypes (Ismail and Hall, 1999; Ram et al., 2012). In the present study, significant genotypic differences have been observed among genotypes for MSI. However, the membrane stability index did not correlate strongly with heat tolerance as few sensitive genotypes (i.e., IPU 99-200) also showed higher membrane stability index. In general, a higher membrane stability index has been observed among tolerant genotypes compared to sensitive genotypes under heat stress in the present investigation. In wheat, genetic variability has been observed for this trait, which could be exploited in the development of a heat tolerant wheat variety (Kumar et al., 2013). Since different physiological stages affect this trait, a particular physiological stage that has maximum correlation of MSI with the heat tolerance is needed to be identified for screening the diverse genotypes under heat stress (Hemantaranjan et al., 2014). Therefore, combinations of different physiological traits can be useful for harnessing higher yield under heat stress conditions as observed in an earlier study (Kumar et al., 2018).

Photosynthetic ETR is a potential physiological trait for screening the heat tolerant genotypes. It determines photosynthetic functionality of plants under high temperature and excessive irradiances. Both are often detrimental for plants due to excess generation of toxic superoxide radicals responsible for damaging the functionality of the photosynthetic system (Allakhverdiev and Murata, 2004). In the present study, photosynthetic ETR was significantly different among studied genotypes. Yamada et al. (1996) reported enhanced physiological efficiency of a genotype under high temperature if it had the ability to maintain higher photosynthetic ETR with increasing PAR. In our study, all heat tolerant genotypes generally showed a curvilinear relationship of photosynthetic ETR with increasing PAR but responses of ETR beyond light saturation (ETR_*max*_) remained significantly higher in highly tolerant genotype-UPU 85-86 (Figure 3) indicating its greater radiation use efficiency even under higher temperature exposure. Whereas the light-saturation point of ETR in highly sensitive one-PKGU-1 was very low and as a result it could not sustain photosynthesis at combined stresses such as high irradiance and high temperature. Thus, sensitive genotypes are more prone to heat stress than tolerant genotypes primarily due to substantial reduction of electron transport and damage of photosystems as reported in earlier studies (Song et al., 2014; Brestic et al., 2016; Chovancek et al., 2019).

The ratio of variable to maximum fluorescence (Fv/Fm), which is known as quantum yield, and the minimal fluorescence (F_0_) show their correlation with heat tolerance (Yamada et al., 1996). These parameters are associated with photosystem II (PSII) and carbon fixation. Heat stress affects the photosynthesis process due to inhibition of the activity of PSII (Camejo et al., 2005; Allakhverdiev et al., 2008; Yamamoto, 2016). In winter wheat significant differences among genotypes have been observed for thermostability of PSII and its acclimation effects on PSII photochemical efficiency (Brestic et al., 2012). However, in another study, high temperature stress also affected PSI due to a non-stomatal limitation of photosynthesis by decreasing the activity of rubisco and other parameters of photochemistry (Salvucci and Crafts-Brandner, 2004; Chovancek et al., 2019). In the present study, decreasing the variable fluorescence (F_*V*_ = Fm – F_0_) leads to decrease in the quantum yield (Fv/Fm) due to inhibition of PSII under heat stress (Kumar et al., 2018). However, quantum yield varied among the studied genotypes and most of the heat tolerant urdbean genotypes had higher quantum yield when grown under high temperature (Figure 4). This indicates that heat tolerant genotypes have superior photosynthetic activity under stress than heat sensitive ones. This could be due to increased activity of antioxidative enzymes SOD and peroxidase, the inherent ability to have higher membrane stability, higher chlorophyll retention capacity, or development of certain compounds in heat tolerant genotypes that protect PSII as reported in earlier studies (Murata et al., 2012).

Different fluorescence parameters were recorded to study the effect of temperature on the photosynthetic activities in two contrasting genotypes having different sensitivity to heat stress. Initial fluorescence intensity (F_0_) measured in the dark-adapted state, when all PSII reaction centers are open, has been used as a thermo-injury index. The increase in the F_0_ in sensitive genotype PKGU-1 as shown in the image (Figure 4) was evident from the color code bar toward the higher side as well as its corresponding numerical value (Table 11). This sudden change in F_0_ is associated with photosynthetic membranes that had suffered irreversible injury. These findings have been supported further from earlier reports by Georgieva and Yordanov (1993). In contrast, heat shocked leaf of tolerant genotype had much lower values of initial fluorescence (F_0_). The maximum F_*m*_ and variable fluorescence showed no significant difference between sensitive and tolerant genotypes. However, quantum yield (F_*v*_/F_*m*_) image and its numerical values were distinctly different, suggesting that altered quantum yield was largely affected by initial fluorescence (F_0_). The higher thermal injury or rise of F_0_ was observed in the sensitive genotype as compared to the tolerant ones.

The significant decrease in F_*v*_/F_*m*_ at high temperature in sensitive genotype (PKGU-1) indicated that plants were under severe stress and that the photochemical efficiency of PSII was severely impaired. This revealed that high temperature significantly affected the photochemistry of PSII leading to photoinhibition (Baker and Rosenqvist, 2004). Furthermore, the sharp decrease in the F_*v*_/F_*m*_ at high temperature was due to the increase in F_0_ under the stress condition. Our results are consistent with earlier reports indicating the decline in F_*v*_/F_*m*_ that involves an increase in F_0_ (Yamada et al., 1996).

The fluorescence parameters such as maximum fluorescence (F_*m*_), quantum yield (F_*v*_/F_*m*_), and minimal fluorescence (F_0_) during light to dark transition phases demonstrate the potential ability of a photosynthetic system to recover to normal values that were observed before the illumination by actinic light. In the present study, the decrease in the effective quantum yield F_*v*_/F_*m*_ was more pronounced in heat-shocked leaves of the sensitive genotype exposed to light condition compared to the tolerant ones which could likely to be due to photoinhibition of PSII associated with increase in F_0_. The photoinhibition is often reversible during light to dark transition but it depends on sensitivity of genotype to heat stress and hence recovery might be delayed. The heat tolerant genotype showed complete recovery in F_*v*_/F_*m*_ in the dark after 500 s, suggesting that reversible changes of photosystems occurred during continuous illumination up to 250 s (Figure 5). However, in the case of sensitive ones, it appeared to undergo an irreversible change for a longer period and could not recover in the dark phase even after 500 s (Figure 5). The delayed recovery of F_*v*_/F_*m*_ could likely be associated with major conformational changes in photosystems to operate in a normal manner.

Maximum decrease in quantum yield indicates damage to the photosynthetic apparatus of the plants (Van der Westhuizen et al., 2020). Many studies have reported variation in the tolerance to high-temperature stress among genotypes of wheat, chickpea, lentil, and mungbean on the basis of pollen sterility, seed abortion, maintenance of photosynthesis, chlorophyll content, and an extended grain-filling duration at elevated temperatures (Viswanathan and Khanna-Chopra, 2001; Tahir and Nakata, 2005; Hays et al., 2007; Krishnamurthy et al., 2011; Kumar et al., 2018; Basu et al., 2019). Heat stress sensitivity of photosynthesis (Singh and Thakur, 2018) due to the inactivation of photosystem II (PSII) (Rustioni et al., 2015) leads to the decrease in variable chlorophyll fluorescence (F_*v*_). This is the most thermolabile component of the photosynthetic electron transport chain (Camejo et al., 2005). Therefore, the detection and quantification of temperature-induced changes in the photosynthetic apparatus is an important tool to distinguish genotypes for their heat stress tolerance (Baker and Rosenqvist, 2004). In the present investigation, significant increase in quantum yield of non-regulated energy dissipation was also observed in the highly heat sensitive genotype PKGU-1 (Table 11). Moreover, more time was required toward quenching of maximum fluorescence F_*m*_ (i.e., delay in quenching of F_*m*_) and high values of quenching of F_0_ indicated severe photo-inactivation of PS II in the sensitive genotype (PKGU-1). This was in complete agreement to the fact that greater thermo-tolerance is associated with faster recovery of photo-damage to PSII. Therefore, rapid overnight recovery of photo-inhibition was observed in tolerant genotype UPU 85-86 (Figure 6 and Table 12).

The qualitative analysis was done to demonstrate *in vivo* visualization of oxidants such as superoxide radicals and hydrogen peroxides, which clearly elucidated the differences in enzymatic activities of superoxide dismutase (SOD) and peroxidase (POX) in heat shocked leaves of extreme heat tolerant and sensitive genotypes (Figure 7). The presence of blue crystalline formazan deposits and dark brown precipitates indicated low activities of SOD and POX enzymes in the sensitive genotype. As a result, the sensitive genotype failed to scavenge the harmful radicals, which caused damage to membranes due to heat stress.

Further, in the case of SSR marker data based dendrogram, a highly heat tolerant genotype (UPU 85-86) was distinctly clustered from the highly heat sensitive genotype (PKGU-1). Heat tolerance being a trait governed by several genes, it becomes very difficult to categorize them solely based on SSR markers unless the markers are highly linked to the heat tolerance trait. Since the primers were designed based on their relevance to abiotic stress tolerance like drought, salinity, and so on, in addition to heat tolerance, the clustering based on their amplification profiles holds importance. The dendrogram obtained by Sun et al. (2015) in tall fescue also showed the heat tolerant genotypes to be strewn across the dendrogram. In addition, a low correlation was found between morpho-physiological heat tolerance traits and SSR markers by the Mantel test (data not shown). The identified genetically diverse and high temperature tolerant lines would be useful in designing breeding programs for developing heat stress tolerance in urdbean.

## Conclusion

Based on field evaluation of 97 urdbean genotypes over two locations under two different growing conditions, a panel of heat tolerant and sensitive genotypes was identified which were stable in yield. Genotypic differences existed for physiological traits like leaf NBI, chlorophyll (SPAD), epidermal flavanols and anthocyanin contents among the tested heat tolerant and sensitive genotypes. The genotypic variation in the membrane stability was evident, which defined the variation in the heat tolerance but to a lesser extent. The high antioxidant activities were shown by heat tolerant genotype (UPU 85-86) explaining their role for scavenging superoxide radicals (ROS) protecting delicate membranes from oxidative damage. Perhaps the higher photosynthetic activities including ETR, quantum yield, and lesser photoinhibition as observed in the heat tolerant genotype UPU 85-86 are associated with inherent stable membranes and higher expression of antioxidative enzymes during exposure to high temperature enabling the plant to maintain optimum functionality. Molecular characterization further pinpointed genetic differences between heat tolerant (UPU 85-86) and heat sensitive genotypes (PKGU-1).

## Data Availability Statement

The original contributions presented in the study are included in the article/Supplementary Material, further inquiries can be directed to the corresponding author/s.

## Author Contributions

DS conceived the idea, conducted the experiments, analyzed data, and wrote the draft manuscript. PB contributed to the writing, reviewing, editing, physiological data, and analysis of experimental data. JS and PD carried out the molecular work. JK conceived the idea and wrote the drafted manuscript. SaG, MP, SG, and NP reviewed and edited the manuscript. VK contributed to the antioxidant profile of two urdbean genotypes. PS conducted the field trials. All authors contributed to the article and approved the submitted version.

## Conflict of Interest

The authors declare that the research was conducted in the absence of any commercial or financial relationships that could be construed as a potential conflict of interest.

## Publisher’s Note

All claims expressed in this article are solely those of the authors and do not necessarily represent those of their affiliated organizations, or those of the publisher, the editors and the reviewers. Any product that may be evaluated in this article, or claim that may be made by its manufacturer, is not guaranteed or endorsed by the publisher.

## References

[B1] Abdel-Aal E. S. M. Abou-Arab A. A. Gamel T. H. Hucl P. Young J. C. Rabalski I. (2008). Fractionation of blue wheat anthocyanin compounds and their contribution to antioxidant properties. *J. Agric. Food Chem.* 56 11171–11177. 10.1021/jf802168c 19007238

[B2] Allakhverdiev S. I. Murata N. (2004). Environmental stress inhibits the synthesis de novo of proteins involved in the photodamage–repair cycle of Photosystem II in Synechocystis sp. PCC 6803. *Biochim. Biophys. Acta BBA.Bioenerget.* 1657 23–32. 10.1016/j.bbabio.2004.03.003 15238209

[B3] Allakhverdiev S. I. Kreslavski V. D. Klimov V. V. Los D. A. Carpentier R. Mohanty P. (2008). Heat stress: an overview of molecular responses in photosynthesis. *Photosyn. Res.* 98 541–550. 10.1007/s11120-008-9331-0 18649006

[B4] Aravind J. Mukesh Sankar S. Wankhede D. P. Kaur V. (2020). *augmentedRCBD: Analysis of augmented randomised complete block designs.* R package version 0.1.*2.* ICAR-NBPGR, New Delhi

[B5] Argyris J. Dahal P. Hayashi E. Still D. W. Bradford K. J. (2008). Genetic variation for lettuce seed thermoinhibition is associated with temperature-sensitive expression of abscisic acid, gibberellin, and ethylene biosynthesis, metabolism, and response genes. *Plant Physiol.* 148 926–947. 10.1104/pp.108.125807 18753282 PMC2556833

[B6] Ashraf M. F. M. R. Foolad M. R. (2007). Roles of glycine betaine and proline in improving plant abiotic stress resistance. *Environ. Exp. Bot.* 59 206–216. 10.1016/j.envexpbot.2005.12.006

[B7] Asseng S. Ewert F. Martre P. Rötter R. P. Lobell D. B. Cammarano D. (2015). Rising temperatures reduce global wheat production. *Nat. Clim. Chang.* 5 143–147. 10.1038/nclimate2470

[B8] Baker N. R. Rosenqvist E. (2004). Applications of chlorophyll fluorescence can improve crop production strategies: an examination of future possibilities. *J. Exp. Bot.* 55 1607–1621. 10.1093/jxb/erh196 15258166

[B9] Basu P. S. Pratap A. Gupta S. Sharma K. Tomar R. Singh N. P. (2019). Physiological traits for shortening crop duration and improving productivity of greengram (*Vigna radiata* L. Wilczek) under high temperature. *Front. Plant Sci.* 10:1508. 10.3389/fpls.2019.01508 31867025 PMC6904351

[B10] Bhandari K. Sharma K. D. Rao B. H. Siddique K. H. Gaur P. Agrawal S. K. (2017). Temperature sensitivity of food legumes: a physiological insight. *Acta Physiol. Plant.* 39:68. 10.1007/s11738-017-2361-5

[B11] Blum A. Ebercon A. (1981). Cell membrane stability as a measure of drought and heat tolerance in wheat. *Crop Sci.* 21 43–47. 10.2135/cropsci1981.0011183X002100010013x

[B12] Blum A. Klueva N. Nguyen H. T. (2001). Wheat cellular thermotolerance is related to yield under heat stress. *Euphytica* 117 117–123. 10.1023/A:1004083305905

[B13] Brestic M. Zivcak M. Kalaji H. M. Carpentier R. Allakhverdiev S. I. (2012). Photosystem II thermostability in situ: environmentally induced acclimation and genotype-specific reactions in *Triticum aestivum* L. *Plant Physiol. Biochem.* 57 93–105. 10.1016/j.plaphy.2012.05.012 22698752

[B14] Brestic M. Zivcak M. Kunderlikova K. Allakhverdiev S. I. (2016). High temperature specifically affects the photoprotective responses of chlorophyll b-deficient wheat mutant lines. *Photosyn. Res.* 130 251–266. 10.1007/s11120-016-0249-7 27023107

[B15] Camejo D. Rodríguez P. Morales M. A. Dell’Amico J. M. Torrecillas A. Alarcón J. J. (2005). High temperature effects on photosynthetic activity of two tomato cultivars with different heat susceptibility. *J. Plant Physiol.* 162 281–289. 10.1016/j.jplph.2004.07.014 15832680

[B16] Castellarin S. D. Pfeiffer A. Sivilotti P. Degan M. Peterlunger E. Di Gaspero G. (2007). Transcriptional regulation of anthocyanin biosynthesis in ripening fruits of grapevine under seasonal water deficit. *Plant Cell Environ.* 30 1381–1399. 10.1111/j.1365-3040.2007.01716.x 17897409

[B17] Challinor A. Wheeler T. Garforth C. Craufurd P. Kassam A. (2007). Assessing the vulnerability of food crop systems in Africa to climate change. *Clim. Change* 83 381–399. 10.1007/s10584-007-9249-0

[B18] Chitra U. Singh U. Rao P. V. (1996). Phytic acid, in vitro protein digestibility, dietary fiber, and minerals of pulses as influenced by processing methods. *Plant Foods Hum. Nutr.* 49 307–316. 10.1007/BF01091980 8983057

[B19] Chovancek E. Zivcak M. Botyanszka L. Hauptvogel P. Yang X. Misheva S. (2019). Transient heat waves may affect the photosynthetic capacity of susceptible wheat genotypes due to insufficient photosystem I photoprotection. *Plants* 8:282. 10.3390/plants8080282 31408991 PMC6724146

[B20] Christensen T. H. Zhang Z. Wei Y. Collinge D. B. (1997). Subcellular localization of H2O2 in plants. H2O2 accumulation in papillae and hypersensitive response during the barley—powdery mildew interaction. *Plant J.* 11 1187–1194.

[B21] Dellaporta S. L. Wood J. Hicks J. B. (1983). A plant DNA minipreparation: version II. *Plant Mol. Biol. Rep.* 1 19–21. 10.1007/BF02712670

[B22] Dhanda S. S. Munjal R. (2006). Inheritance of cellular thermotolerance in bread wheat. *Plant Breed.* 125 557–564. 10.1111/j.1439-0523.2006.01275.x

[B23] Ehlers J. D. Hall A. E. (1998). Heat tolerance of contrasting cowpea lines in short and long days. *Field Crops Res.* 55 11–21. 10.1016/S0378-4290(97)00055-5

[B24] Fischer R. A. Maurer R. (1978). Drought resistance in spring wheat cultivars. 1. Grain-yield responses. *Aust. J. Agric. Res.* 29 897–912. 10.1071/AR9780897

[B25] Frahry G. Schopfer P. (2001). NADH-stimulated, cyanide-resistant superoxide production in maize coleoptiles analyzed with a tetrazolium-based assay. *Planta* 212 175–183. 10.1007/s004250000376 11216837

[B26] Frutos E. Galindo M. P. Leiva V. (2014). An interactive biplot implementation in R for modeling genotype-by-environment interaction. *Stoch. Environ. Res. Risk Assess.* 28 1629–1641.

[B27] Gabriel K. R. (1971). The biplot graphic display of matrices with application to principal component analysis. *Biometrika* 58 453–467. 10.1093/biomet/58.3.453

[B28] Gaur P. M. Samineni S. Thudi M. Tripathi S. Sajja S. B. Jayalakshmi V. (2019). Integrated breeding approaches for improving drought and heat adaptation in chickpea (*Cicer arietinum* L.). *Plant Breed.* 138 389–400. 10.1111/pbr.12641

[B29] Georgieva K. Yordanov I. (1993). Temperature dependence of chlorophyll fluorescence parameters of pea seedlings. *J. Plant Physiol.* 142 151–155. 10.1016/S0176-1617(11)80955-7

[B30] Gowda M. B. (2008). *Genetic enhancement of Dolichos bean through integration of conventional breeding and molecular approaches.* Final Project Report. Witney: The Kirkhouse Trust.

[B31] Hays D. B. Do J. H. Mason R. E. Morgan G. Finlayson S. A. (2007). Heat stress induced ethylene production in developing wheat grains induces kernel abortion and increased maturation in a susceptible cultivar. *Plant Sci.* 172 1113–1123. 10.1016/j.plantsci.2007.03.004

[B32] Hemantaranjan A. Bhanu A. N. Singh M. N. Yadav D. K. Patel P. K. Singh R. (2014). Heat stress responses and thermotolerance. *Adv. Plants Agric. Res.* 1 1–10. 10.15406/apar.2014.01.00012

[B33] Ismail A. M. Hall A. E. (1999). Reproductive-stage heat tolerance, leaf membrane thermostability and plant morphology in cowpea. *Crop Sci.* 39 1762–1768. 10.2135/cropsci1999.3961762x

[B34] Jaccard P. (1908). Nouvelles recherches sur la distribution florale. *Bull.Soc. Vaudoise des Sci. Nat.* 44 223–270.

[B35] Krishnamurthy L. Gaur P. M. Basu P. S. Chaturvedi S. K. Tripathi S. Vadez V. (2011). Large genetic variation for heat tolerance in the reference collection of chickpea (*Cicer arietinum* L.) germplasm. *Plant Genet. Resour.* 9 59–69. 10.1017/S1479262110000407

[B36] Kumar J. Basu P. S. Gupta S. Dubey S. Gupta D. S. Singh N. P. (2018). Physiological and molecular characterisation for high temperature stress in *Lens culinaris*. *Funct. Plant Biol.* 45 474–487. 10.1071/FP17211 32290986

[B38] Kumar S. Kumari P. Kumar U. Grover M. Singh A. K. Singh R. (2013). Molecular approaches for designing heat tolerant wheat. *J. Plant Biochem. Biotechnol.* 22 359–371. 10.1007/s13562-013-0229-3

[B39] Murata N. Allakhverdiev S. I. Nishiyama Y. (2012). The mechanism of photoinhibition in vivo: re-evaluation of the roles of catalase, α-tocopherol, non-photochemical quenching, and electron transport. *Biochim. Biophys. Acta BBA Bioenerget.* 1817 1127–1133. 10.1016/j.bbabio.2012.02.020 22387427

[B40] Nayyar H. Gaur P. M. Kumar S. Singh S. Bindumadhava H. Nair R. M. (2017). “How rising temperatures would be detrimental for cool and warm-season food legumes,” in *Proceedings of the InterDrought-V, February 21-25, 2017*, (Hyderabad: HICC).

[B41] Paliwal R. Röder M. S. Kumar U. Srivastava J. P. Joshi A. K. (2012). QTL mapping of terminal heat tolerance in hexaploid wheat (*T. aestivum* L.). *Theor. Appl. Genet.* 125 561–575. 10.1007/s00122-012-1853-3 22476874

[B42] Pandey G. C. Mamrutha H. M. Tiwari R. Sareen S. Bhatia S. Siwach P. (2015). Physiological traits associated with heat tolerance in bread wheat (*Triticum aestivum* L.). *Physiol. Mol. Biol. Plants* 21 93–99. 10.1007/s12298-014-0267-x 25648644 PMC4312329

[B43] Pootakham W. Nawae W. Naktang C. Sonthirod C. Yoocha T. Kongkachana W. (2021). A chromosome-scale assembly of the black gram (*Vigna mungo*) genome. *Mol. Ecol. Resour.* 21 238–250. 10.1111/1755-0998.13243 32794377

[B44] Project Coordinator’s Report (2019). *All India Coordinated Research Project on Mungbean, Urdbean, Lentil, Lathyrus, Field Pea.* Kanpur: Indian Institute of Pulses Research.

[B45] R Core Team (2019). *R: a Language and Environment for Statistical computing.* Vienna: R Foundation for Statistical Computing.

[B46] Raizada A. (2020). *Development of genic-markers for yellow mosaic virus and bruchid resistance traits in blackgram (Vigna mungo (L.) Hepper).* Ph.D. Thesis. Mumbai: Homi Bhabha National Institute, 214–265.

[B47] Raizada A. Souframanien J. (2019). Transcriptome sequencing, de novo assembly, characterisation of wild accession of blackgram (*Vigna mungo* var. silvestris) as a rich resource for development of molecular markers and validation of SNPs by high resolution melting (HRM) analysis. *BMC Plant Biol.* 19:358. 10.1186/s12870-019-1954-0 31419947 PMC6697964

[B48] Ram B. Singh B. K. Singh M. Singh V. V. Chauhan J. S. (2012). *Physiological And Molecular Characterization Of Indian Mustard (b. Juncea l.) Genotypes for High Temperature Tolerance.* London: Crop Improvement (ICSA), 5–6.

[B49] Rohlf F. J. (1998). *NTSYS-Pc: Numerical taxonomy and Multivariate Analysis System, Version 2.0.* Setauket: Exeter publications.

[B50] Roy S. J. Tucker E. J. Tester M. (2011). Genetic analysis of abiotic stress tolerance in crops. *Curr. Opin. Plant Biol.* 14 232–239. 10.1016/j.pbi.2011.03.002 21478049

[B51] Rustioni L. Milani C. Parisi S. Failla O. (2015). Chlorophyll role in berry sunburn symptoms studied in different grape (*Vitis vinifera* L.) cultivars. *Sci. Hortic.* 185 145–150. 10.1016/j.scienta.2015.01.029

[B52] Salvucci M. E. Crafts-Brandner S. J. (2004). Relationship between the heat tolerance of photosynthesis and the thermal stability of *Rubisco activase* in plants from contrasting thermal environments. *Plant Physiol.* 134 1460–1470. 10.1104/pp.103.038323 15084731 PMC419822

[B53] Scafaro A. P. Haynes P. A. Atwell B. J. (2010). Physiological and molecular changes in *Oryza meridionalis* Ng., a heat-tolerant species of wild rice. *J. Exp. Bot.* 61 191–202. 10.1093/jxb/erp294 19819927 PMC2791120

[B54] Schreiber U. Bilger W. (1987). “Rapid assessment of stress effects on plant leaves by chlorophyll fluorescence measurements,” in *Plant Response To Stress*, eds Tenhunen J. D. Catarino F. M. Lange O. L. Oechel W. C. (Berlin: Springer), 27–53. 10.1007/978-3-642-70868-8_2

[B55] Sen Gupta D. Singh U. Kumar J. Shivay Y. S. Dutta A. Sharanagat V. S. (2020). Estimation and multi-variate analysis of iron and zinc concentration in a diverse panel of urdbean (Vigna mungo L. Hepper) genotypes grown under differing soil conditions. *J. Food Comp. Anal.* 93:103605. 10.1016/j.jfca.2020.103605

[B56] Sikder S. Ahmed J. U. Hossain T. (2001). Heat tolerance and relative yield performance of wheat varieties under late seeded conditions. *Indian J. Agric. Res.* 35 141–148.

[B57] Singh J. Thakur J. K. (2018). “Photosynthesis and abiotic stress in plants,” in *Biotic And Abiotic Stress Tolerance in Plants*, ed. Vats S. (Singapore: Springer), 27–46. 10.1007/978-981-10-9029-5_2

[B58] Sita K. Sehgal A. HanumanthaRao B. Nair R. M. Vara Prasad P. V. Kumar S. (2017). Food legumes and rising temperatures: effects, adaptive functional mechanisms specific to reproductive growth stage and strategies to improve heat tolerance. *Front. Plant Sci.* 8:1658. 10.3389/fpls.2017.01658 29123532 PMC5662899

[B59] Song Y. Chen Q. Ci D. Shao X. Zhang D. (2014). Effects of high temperature on photosynthesis and related gene expression in poplar. *BMC Plant Biol.* 14:111. 10.1186/1471-2229-14-111 24774695 PMC4036403

[B60] Souframanien J. Raizada A. Dhanasekar P. Suprasanna P. (2020). Draft genome sequence of the pulse crop blackgram [*Vigna mungo* (L.) Hepper] reveals potential R-genes. *Sci. Rep.* 11:11247. 10.1038/s41598-021-90683-9 34045617 PMC8160138

[B61] Sun X. Xie Y. Bi Y. Liu J. Amombo E. Hu T. (2015). Comparative study of diversity based on heat tolerant-related morpho-physiological traits and molecular markers in tall fescue accessions. *Sci. Rep.* 5 1–14. 10.1038/srep18213 26666506 PMC4678371

[B62] Tahir I. S. A. Nakata N. (2005). Remobilization of nitrogen and carbohydrate from stems of bread wheat in response to heat stress during grain filling. *J. Agron. Crop Sci.* 191 106–115. 10.1111/j.1439-037X.2004.00127.x

[B63] Tewari A. K. Tripathy B. C. (1998). Temperature-stress-induced impairment of chlorophyll biosynthetic reactions in cucumber and wheat. *Plant Physiol.* 117 851–858. 10.1104/pp.117.3.851 9662527 PMC34939

[B64] Van der Westhuizen M. M. Oosterhuis D. M. Berner J. M. Boogaers N. (2020). Chlorophyll a fluorescence as an indicator of heat stress in cotton (*Gossypium hirsutum* L.). *South Afr. J. Plant Soil* 37 116–119. 10.1080/02571862.2019.1665721

[B65] Venkatesha S. C. Gowda M. B. Mahadevu P. Rao A. M. Kim D. J. Ellis T. H. N. (2007). Genetic diversity within Lablab purpureus and the application of gene-specific markers from a range of legume species. *Plant Genet. Resour.* 5 154–171. 10.1017/S1479262107835659

[B66] Viswanathan C. Khanna-Chopra R. (2001). Effect of heat stress on grain growth, starch synthesis and protein synthesis in grains of wheat (*Triticum aestivum* L.) varieties differing in grain weight stability. *J. Agron. Crop Sci.* 186 1–7. 10.1046/j.1439-037x.2001.00432.x

[B67] Xu P. Wu X. Wang B. Liu Y. Ehlers J. D. Close T. J. (2011). A SNP and SSR based genetic map of asparagus bean (*Vigna. unguiculata* ssp. sesquipedialis) and comparison with the broader species. *PLoS One* 6:e15952. 10.1371/journal.pone.0015952 21253606 PMC3017092

[B68] Yamada M. Hidaka T. Fukamachi H. (1996). Heat tolerance in leaves of tropical fruit crops as measured by chlorophyll fluorescence. *Sci. Hortic.* 67 39–48. 10.1016/S0304-4238(96)00931-4

[B69] Yamamoto Y. (2016). Quality control of photosystem II: the mechanisms for avoidance and tolerance of light and heat stresses are closely linked to membrane fluidity of the thylakoids. *Front. Plant Sci.* 7:1136. 10.3389/fpls.2016.01136 27532009 PMC4969305

[B70] Yan W. (2001). GGEbiplot—A Windows application for graphical analysis of multienvironment trial data and other types of two-way data. *Agron. J.* 93 1111–1118. 10.2134/agronj2001.9351111x

[B71] Yan W. Hunt L. A. Sheng Q. Szlavnics Z. (2000). Cultivar evaluation and mega-environment investigation based on the GGE biplot. *Crop Sci.* 40 597–605. 10.2135/cropsci2000.403597x

